# Design and synthesis of novel main protease inhibitors of COVID-19: quinoxalino[2,1-*b*]quinazolin-12-ones†

**DOI:** 10.1039/d4ra06025c

**Published:** 2024-09-13

**Authors:** Atefeh Tirehdast, Seddigheh Sheikhi-Mohammareh, Hossein Sabet-Sarvestani, Michael G. Organ, Volodymyr Semeniuchenko, Ali Shiri

**Affiliations:** a Department of Chemistry, Faculty of Science, Ferdowsi University of Mashhad Mashhad Iran alishiri@um.ac.irs; b Department of Chemistry and Biomolecular Sciences, Faculty of Science, University of Ottawa Ottawa Canada

## Abstract

The COVID-19 pandemic represents a substantial global challenge, being a significant cause of mortality in numerous countries. Thus, it is imperative to conduct research to develop effective therapies to combat COVID-19. The primary aim of this study is to employ a two-step tandem reaction involving 2,3-dichloroquinoxaline and 2-amino-*N*-substituted benzamides in alkaline media/DMF at an elevated temperature to design and synthesize a series of polycyclic derivatives endowed with quinoxalino[2,1-*b*]quinazolin-12-one framework. Following synthesis, the newly synthesized heterocycles were evaluated for their potential as inhibitors of the main protease of SARS-CoV-2 by means of molecular docking and dynamic simulation techniques. The *in silico* investigation demonstrated that all tested compounds effectively establish stable binding interactions, primarily through multiple hydrogen bonding and hydrophobic interactions, at the active site of the enzyme. These findings offer crucial structural insights that can be employed in future endeavors toward designing potent inhibitors targeting the main protease (M^pro^). Among the investigated compounds, the *p*-tolylamino-substituted quinoxalino[2,1-*b*]quinazolinone derivative exhibited the most promise as an inhibitor of the main protease in COVID-19. Consequently, it warrants further investigation both *in vitro* and *in vivo* to identify it as a prospective candidate for anti-SARS-CoV-2 drug development.

## Abstract 2

Aiming to discover potent inhibitors targeting 3CL^pro^, an efficient procedure has been established for the synthesis of novel quinoxalino[2,1-*b*]quinazolin-12-ones.

## Graphical abstract

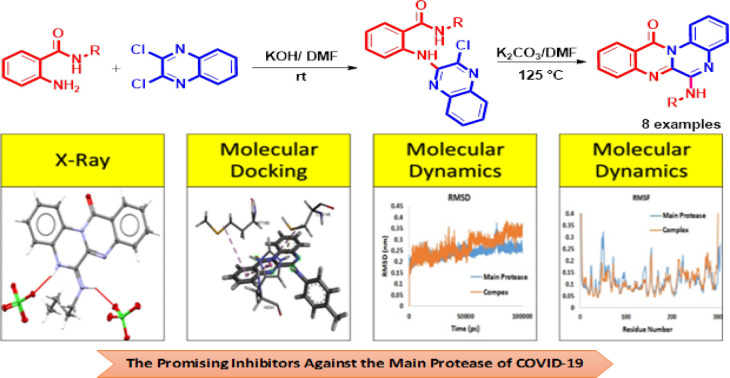

## Introduction

The global pandemic, instigated by Severe Acute Respiratory Syndrome Coronavirus 2 (SARS-CoV-2), has resulted in significant consequences.^1,2^ Since its initial emergence in December 2019, SARS-CoV-2 (the causative agent of COVID-19) has impacted over 676 million individuals worldwide, leading to a mortality rate of 6.93 million, as cited by the World Health Organization on May 24th, 2023. Currently, individuals remain susceptible to recurrent SARS-CoV-2 infections. Despite the development of vaccines by Pfizer, Moderna, and AstraZeneca since 2020, these preventive measures have demonstrated insufficiency in countering the unforeseen mutations of SARS-CoV-2. Consequently, there is a pressing need to advance more effective antiviral therapeutics.^3^ SARS-CoV-2, a member of the β-coronavirus genus, is an RNA virus distinguished by its positive single-stranded genome. The replication process of this virus involves the participation of two proteolytic enzymes: the main protease (M^pro^) and papain-like protease (PL^pro^).^4–6^ M^pro^, also known as 3-chymotrypsin-like protease (3CL^pro^), shows significant promise as a target for therapeutic intervention against SARS-CoV-2 due to its noteworthy conservation across β-coronaviruses and limited similarity to human proteases.^7,8^ Moreover, studies have revealed substantial sequence similarity among M^pro^ proteins within the coronavirus group,^9–12^ implying that antivirals targeting SARS-CoV-2 M^pro^ may possess the potential to exhibit broad-spectrum effectiveness against various coronaviruses in the future.^13,14^ The catalytic dyad of 3CL^pro^, located within the active site between domains I (residues 8–101) and II (residues 102–184), comprises Cys145 and His41 amino acid residues. This dyad serves a crucial function in facilitating the replication of the viral genome.^15–17^ As a result, two separate classes of potent inhibitors, including both peptidomimetic and nonpeptidic inhibitors, have been identified as viable approaches in combating the swift spread of the COVID-19 pandemic.^18–22^

**Fig. 1:**
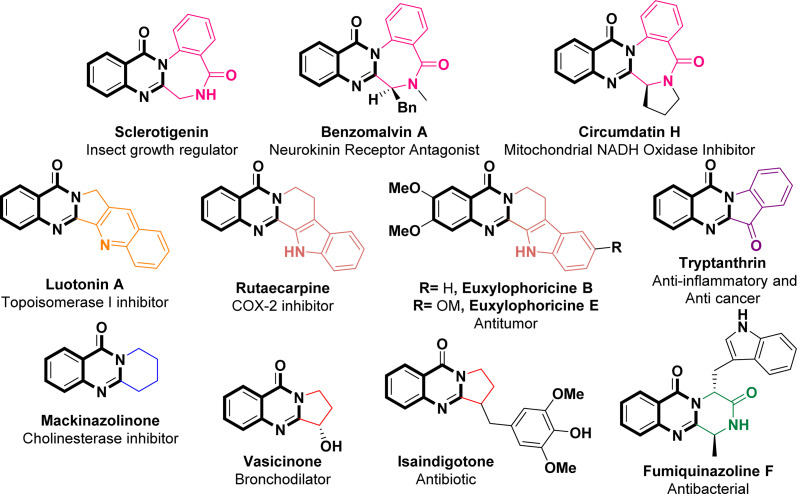
Representative natural products and drugs with 2,3-fused quinazolinone polycyclic skeletons.

As per FDA data covering the period from 2015 to 2020, it has been reported that approximately 88% of the small-molecule drugs approved contain heterocyclic skeletons.^23^ Quinazolinone, a nitrogen-containing heterocyclic building block that acts as a pharmacophore, exhibits a wide array of pharmacological activities spanning anti-inflammatory,^24^ antitumor,^25^ anticonvulsive,^26^ sedative,^27^ antihypertensive,^28^ vasodilatory,^29^ antimicrobial and antibacterial effects,^30^ thereby highlighting its potential for advancement within the domain of drugs and medicine. The literature survey revealed that 2-aminoquinazolin-4-(3*H*)-ones possess potent antiviral effects against both SARS-CoV-2 and MERS-CoV.^31–33^ In particular, 7-chloro-2-((3,5-dichlorophenyl)amino)quinazolin-4(3*H*)-one displayed significant efficacy (with an IC_50_ value of 0.23 μM) in combating SARS-CoV-2 when tested in a Vero cell assay. Additionally, the mentioned compound exhibited no toxicity and demonstrated favorable *in vitro* pharmacokinetic properties including high microsomal stability and minimal binding to hERG channels.^31^ A specific subset of quinazolinone compounds, namely the 2,3-fused quinazolinones, assumes pivotal roles in various pharmaceuticals and natural products, exemplified by Sclerotigenin, Benzomalvin A, Circumdatin H, Luotonin A, Rutaecarpine, Euxylophoricine B, Euxylophoricine E, Tryptanthrin, Mackinazolinone, Vasicinone, Isaindigotone, and Fumiquinazoline F (Fig. 1).^34–36^

Despite the significant efforts made for the synthesis of novel valuable polycyclic quinazolinone-fused structures,^37–39^ there is still a demand for novel methodologies that can efficiently produce diverse quinoxalino[2,1-*b*]quinazolinones. This pursuit poses persistent challenges for organic chemists primarily because of the scarcity of literature on the synthesis of quinoxalino[2,1-*b*]quinazolinones, with only one study found thus far by Pan *et al.* in February 2024. The recently published procedure offers a solution to this gap by employing a cascade radical cyclization strategy, involving the reaction of 3-(2-isocyanophenyl)quinazolin-4(3*H*)-ones with ethers under photocatalytic and metal-free conditions.^40^

Soon after the outbreak of COVID-19, we designed and synthesized several novel heterocyclic architectures and theoretically evaluated their antiviral inhibitory against SARS-CoV-2 M^pro^ in order to contribute to the efforts in tackling the rising threat of fatal coronaviruses.^41,42^ With this background, stemming from our ongoing research interest in the synthesis of novel potentially bioactive heterocyclic scaffolds,^43–52^ and considering the potent applications of quinazolinone-based heterocycles against both SARS-CoV-2 and MERS-CoV,^31–33^ we have developed a novel methodology for synthesizing derivatives of quinoxalino[2,1-*b*]quinazolinones. Computational assessments were carried out to explore the interaction of the synthetic N-heterocyclic compounds with the SARS-CoV-2 M^pro^, utilizing docking and molecular dynamics approaches. These assessments aimed to describe novel nonpeptidic and non-covalent inhibitors of COVID-19 M^pro^ derived from a two-component reaction involving 2,3-dichloroquinoxaline and 2-amino-*N*-substituted benzamides in DMF.

## Results and discussion

**Scheme 1:**
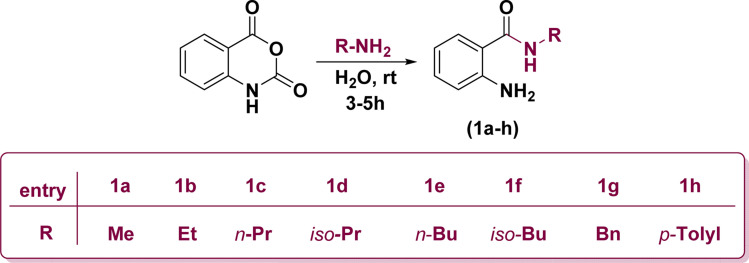
Construction of anthranilamide derivatives.

Initially, the ring opening of isatoic anhydride through the reaction of several primary amines in aqueous media at room temperature resulted in the formation of off-white anthranilamide derivatives (1a–h) (Scheme 1).^53^

**Scheme 2:**
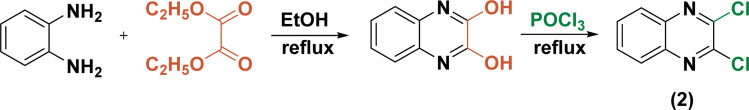
Construction of 2,3-dicholoroquinoxaline.

On the other hand, 2,3-dichloroquinoxaline (2) was also synthetically prepared through the treatment of equimolar amounts of *ortho*-phenylenediamine and diethyloxalate in refluxing ethanol for 6 h followed by chlorination of the obtained quinoxaline-2,3-diol (Scheme 2).^54^

**Scheme 3:**
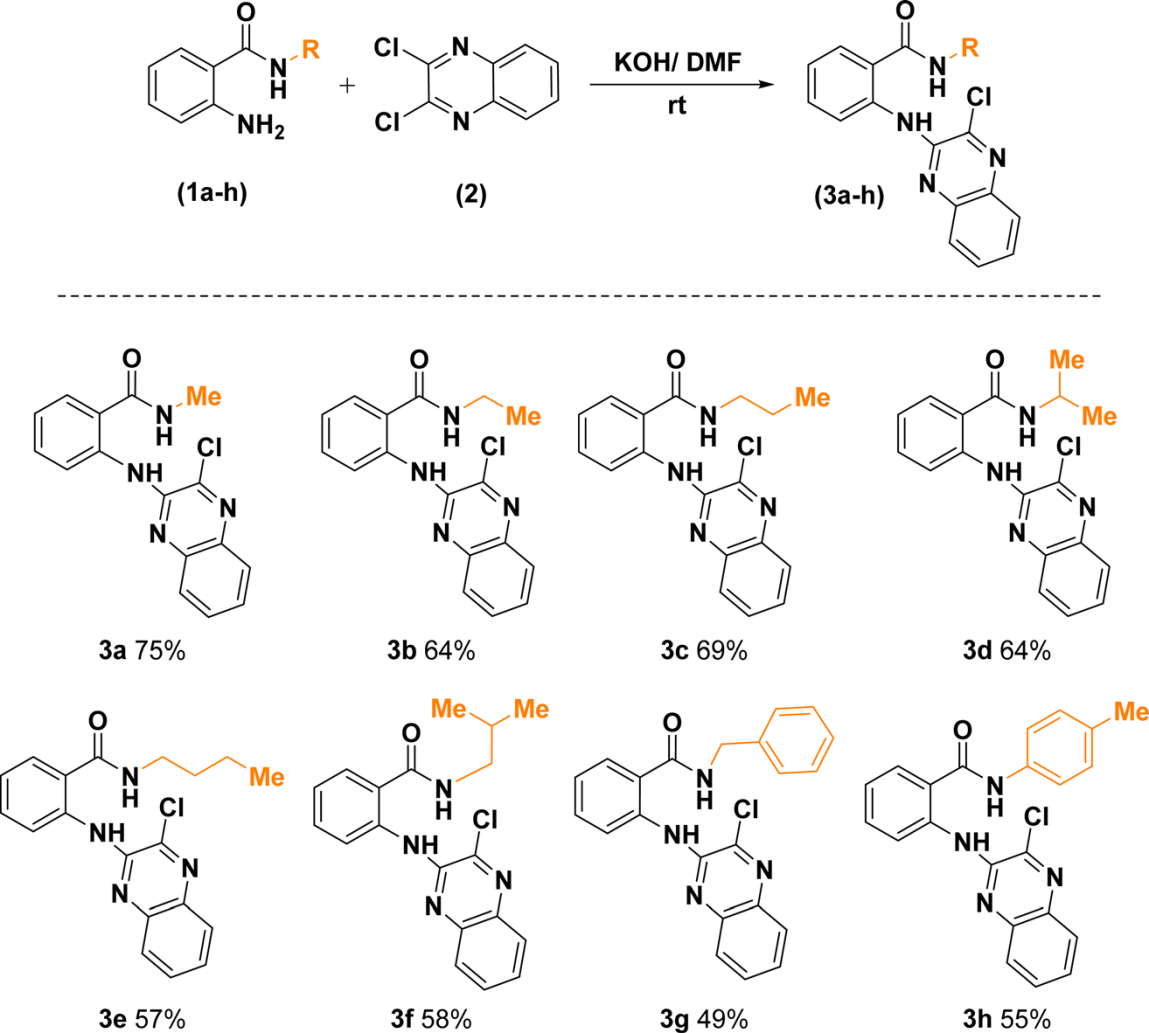
Synthetic route for the construction of 2-((3-chloroquinoxalin-2-yl)amino)-*N*-substituted benzamides.

Replacement of one of the chlorine atoms of the substrate (2) with –NH_2_ group of compounds (1a–h) through S_N_Ar amination afforded quinoxaline derivatives (3a–h) endowed with *ortho*-amino-*N*-alkyl/phenyl benzamide substituents appended in C-2 position. These substitution reactions proceeded at room temperature in the presence of potassium hydroxide dissolved in dimethylformamide (Scheme 3).

**Scheme 4:**
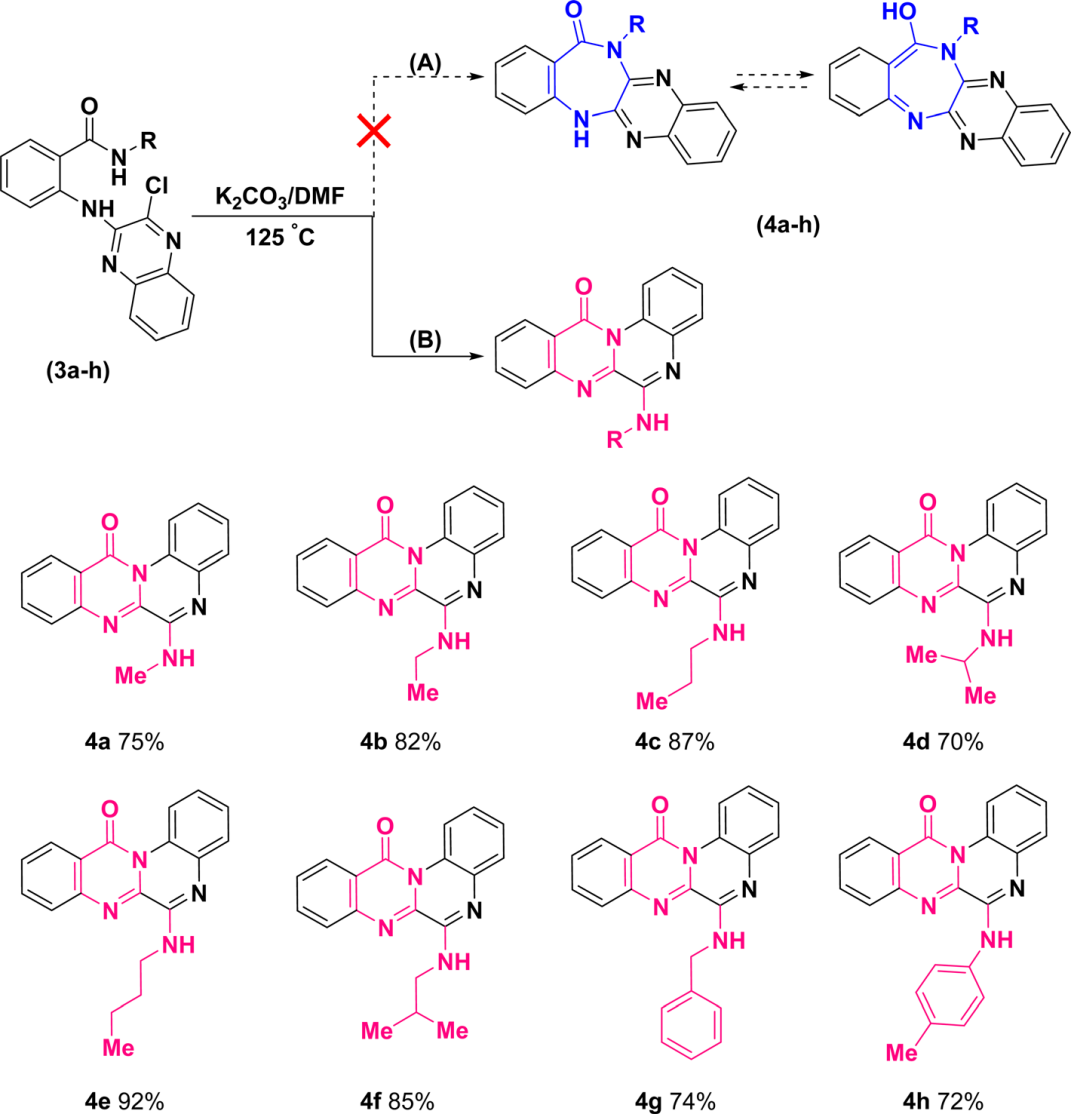
Synthetic route for the construction of 6-(alkylamino)-12*H*-quinoxalino[2,1-*b*]quinazolin-12-ones.

Further treatment of di-hetero(aryl) amine compounds (3a–h) with K_2_CO_3_ in DMF medium at elevated temperature facilitated the synthesis of products (4a–h) that displayed a blue shift for amidic C=O stretching band (based on the infrared spectra) as well as chlorine atom removal (according to the mass spectra). These findings which uniformly observed across all the newly synthesized compounds, provided unequivocal establishment for the definite occurrence of heterocyclization. However, direct intramolecular nucleophilic substitution of amidic moiety on C(sp^2^)–Cl of quinoxaline scaffold (pathway (A)) or tandem multistep pathway (B) leading to annulation of diazepinones or pyrimidinones, respectively, is open to dispute (Scheme 4).

The structural assignments of all the newly synthesized compounds (4a–h) were corroborated by spectroscopic and microanalytical data. Although the formation of both structural isomers (A) and (B) was predicted, the experimental results revealed the formation of only one isomer, either (A) or (B). In the ^1^H-NMR spectra of compounds (4a–h), a broad signal (with slight multiplicity) around *δ* 7.10–7.25 ppm and a singlet signal at *δ* 9.77 ppm, belonging to a D_2_O-exchangeable proton, were observed for compounds (4a–g) and (4h), respectively. Regarding compound (4a), for instance, upon exchange of XH to XD (X: O in isomer (A) or N in isomer (B)) in the presence of D_2_O, the corresponding broad signal at *δ* 7.12 ppm vanished and the doublet signal of methyl protons was transformed into a singlet signal at *δ* 3.21 ppm (S32, ESI†).

This phenomenon can be justified only in the case of a correlation between the exchangeable proton and methyl protons, either in terms of bonding or space. Consequently, ^1^H–^1^H correlation spectroscopy (COSY) (S33, ESI†) and nuclear Overhauser effect spectroscopy (NOESY) (S34, ESI†) were conducted on compound (4a). Resembling the ^1^H-NMR, both of the two-dimensional spectra supported that the aforementioned hydrogen groups were coupled to each other due to the existence of cross-peaks.

**Fig. 2:**
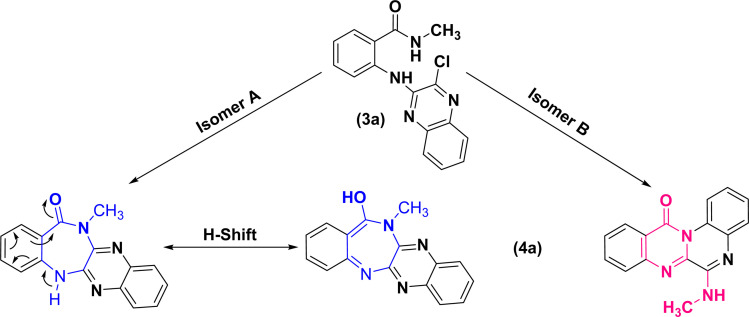
Structural isomeric candidates for annulation of (3a) into (4a).

As 1D and 2D-NMR evidences did not succeed to differentiate between frameworks (A) and (B), the following structures could still be the robust isomeric candidates for cyclization of (3a) into (4a) (Fig. 2).

**Fig. 3:**
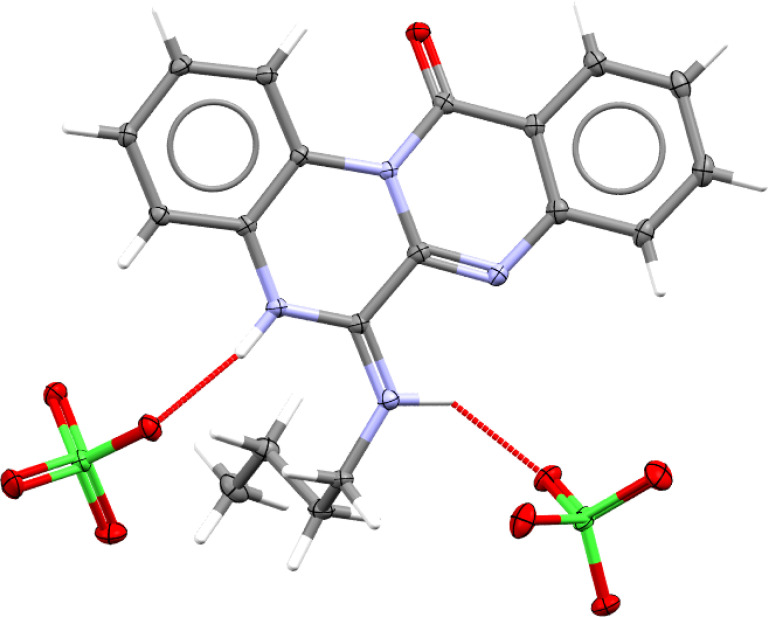
Molecular structure of 4e–perchlorate (CCDC 2357348). Asymmetric unit contains one 4e–H^+^ cation and one ClO_4_^−^ anion, however, hydrogen bonding connects one cation to two anions (as shown here) and *vice versa*.

Eventually, a single crystal X-ray diffraction analysis should be employed to ascertain how cyclization has occurred. In this regard, slow evaporation of (4e)–ethanol solution yielded thin poorly diffracting needles, which allowed solving atoms connectivity, and confirmed the structure (4e). High quality crystals of (4e)–perchlorate salt were grown by slow evaporation from a binary mixture of ethanol–aqueous perchloric acid. Fig. 3 depicts the molecular structure of 4e perchlorate.

**Fig. 4:**
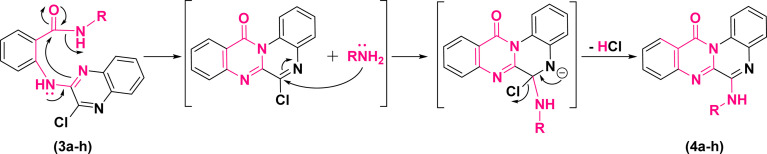
Plausible mechanism for the synthesis of 6-(alkylamino)-12*H*-quinoxalino[2,1-*b*]quinazolin-12-ones.

According to these results, the plausible mechanism for the synthesis of quinoxalino[2,1-*b*]quinazolinone derivatives (4a–h) from (3a–h) has been proposed as a self-explanatory domino pathway. This pathway includes an intramolecular nucleophilic substitution of amidic carbonyl followed by an *in situ* S_N_Ar amination. As depicted in Fig. 4, first, the endocyclic N of quinoxaline moiety of (3a–h) performs a nucleophilic attack on the carbonyl group leading to the cyclization of pyrimidinone. Then, the replacement of chlorine atom with non-isolated nucleophiles (–NH_2_R) accompanied by the elimination of HCl leads to the formation of the corresponding derivatives of (4a–h).

### Computational analyses

Computer-aided drug design (CADD) is a powerful approach that utilizes computational methods and algorithms to expedite and improve the drug discovery and development process. CADD techniques aid in identifying and validating potential drug targets by analyzing biological data and understanding the interactions between proteins, enzymes, receptors, and other molecules involved in disease pathways. This helps researchers pinpoint the most promising targets for therapeutic intervention.

### Methodology

#### Molecular docking

One of the most practical methods for computer-assisted drug design involves molecular docking, which is utilized to determine the optimal positioning of ligands within protein active sites. Molecular docking serves as a pivotal tool for exploring the interplay between ligands and receptors, as well as the interaction dynamics between enzymes and ligands. In drug discovery endeavors, molecular docking is utilized to elucidate structure–activity relationships and to probe the interaction between synthesized compounds and enzyme-active sites. In our investigation, we employed a pre-existing complex form of the three-dimensional crystal structure of the main protease of SARS-CoV-2, containing an inhibitor known as N3, sourced from the RCSB Protein Data Bank (PDB ID: 6LU7), as reported by Jin and colleagues.^8^ Following the removal of the inhibitor and water molecules, any absent atoms were rectified using Chimera and Notepad++.^55^ The refined enzyme structure was then subjected to molecular docking investigations and simulations. The binding site amino acids were identified using the BIOVIA Discovery Studio Visualizer.^56^ Docking calculations were conducted using the AutoDock-Vina software.^57^ Prior to commencing the molecular docking process, polar hydrogen atoms and charges were incorporated into the cleaned enzyme, and the AutoDock Tools were utilized to convert the PDB format to the Partial Charge and Atom Type (PDBQT) format. Additionally, all synthesized molecules underwent optimization using Gaussian 09 software at the B3LYP/6-31G(d,p) level of theory.^58^ Similarly, PDBQT formats of the optimized ligands were generated, taking into account polar hydrogen atoms and Gasteiger charges, using AutoDock Tools. The grid box dimensions applied for all docking investigations were set at 60 Å × 60 Å × 60 Å, centered on the respective geometric center. The Cartesian coordinates of the grid box were aligned with the enzyme at coordinates *x* = −14.212; *y* = 19.133; *z* = 64.83. The energy values of the conformations were determined for 20 geometry conformers using a genetic algorithm known as the Lamarckian algorithm.^59^ In this approach, a population of conformations was generated and ranked based on their binding energy values in kcal mol^−1^. Table 1 depicts the calculated binding affinities and the involved residues, showing that all of the synthesized compounds have lower bending affinity than the N3 ligand as a reference. However (4h) compound possesses the lowest binding affinity which can be considered for the molecular dynamic simulation step.

**Table 1:** Docking results of the synthesized compounds

Compound	Interacting residues	Binding affinity (kcal mol^−1^)
(4a)	His163, Cys145, Met49	−7.7
(4b)	His41, Cys145, Met49, Leu27, Met165	−7.6
(4c)	His163, Cys145, Met49, Leu27, Met165	−7.7
(4d)	Met165, His41, Cys145, Leu27	−7.9
(4e)	His41, Cys145, Leu27, Gln189, Ser144, Gly143, Asn142	−7.8
(4f)	Met165, His41, Cys145, Gln189, Asn142, Gly143, Ser144, Leu27	−8.1
(4g)	Met165, His163, Cys145, Met49, Thr25	**−8.7**
(4h)	Met165, His41, Cys145	**−9.2**
N3(Ref)	Leu287, Leu272, Tyr237, Ala193, Thr169	−6.4

**Fig. 5:**
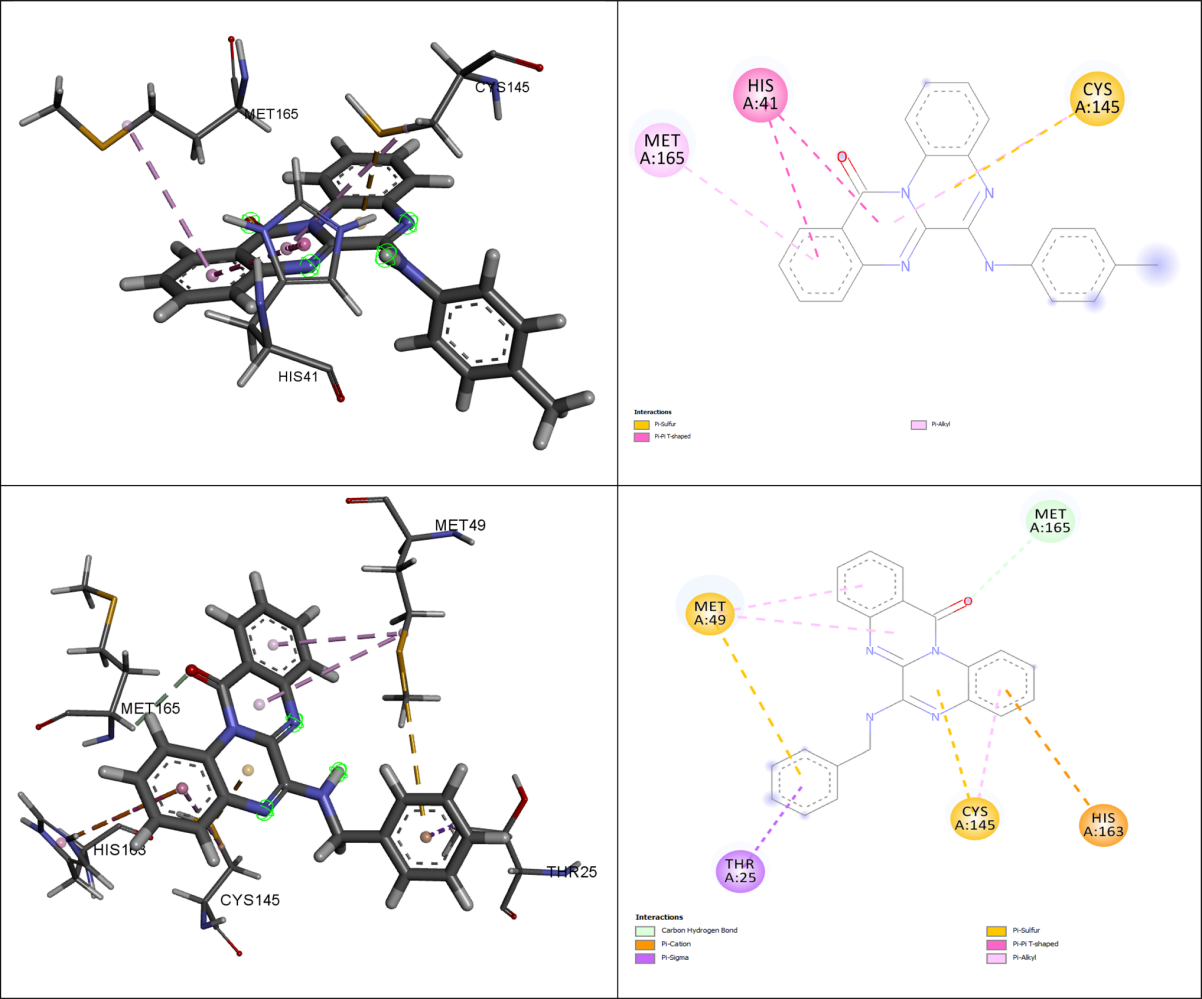
The interacting amino acids for the best-docked synthesized compounds: (4g) (down) and (4h) (up).

Fig. 5 illustrates the interactions of amino acids and the binding modes of compounds (4g) and (4h).

#### Molecular dynamics simulation

The most favorable conformers from the docking analyses of the compound (4h) were selected for molecular dynamic simulations using the AMBER99SB-ILDN force field in Gromacs 2020.^60^ A cubic box filled with simple point charge (SPC)^61^ water molecules was employed to solvate the docked complexes, with Na^+^ or Cl^−^ ions added to maintain overall electrostatic neutrality. Long-range electrostatic interactions were treated using the Particle Mesh Ewald (PME) method, employing a direct space cut-off of 1.2 nm and a Fourier grid spacing of 0.16 nm.^62^ A cutoff distance of 1.2 Å was applied for long-range van der Waals (VDW) energy calculations. Temperature and pressure during the simulation were controlled using a V-rescale thermostat^63^ and Parrinello–Rahman barostat,^64^ respectively, with pressure set to 1 atm and temperature maintained at 298.15 K using a 2 ps time step.

The entire system underwent energy minimization without restraints using the steepest descent algorithm^65^ for 50 000 iteration steps to attain equilibrium. Equilibration was achieved through two distinct simulation steps. Initially, the systems were equilibrated under a constant number of particles, volume, and temperature (*NVT* ensemble)^66^ for 200 ps at 310 K, employing the Verlet scheme to regulate temperature. Subsequently, equilibration continued under a constant number of particles, pressure, and temperature (*NPT* ensemble)^67^ at 300 K for 200 ps, with Berendsen barostat and Verlet scheme^68^ used to control pressure (set to 1 bar) and temperature, respectively. The LINCS algorithm was utilized to manage covalent bond constraints during equilibration.^69^

**Fig. 6:**
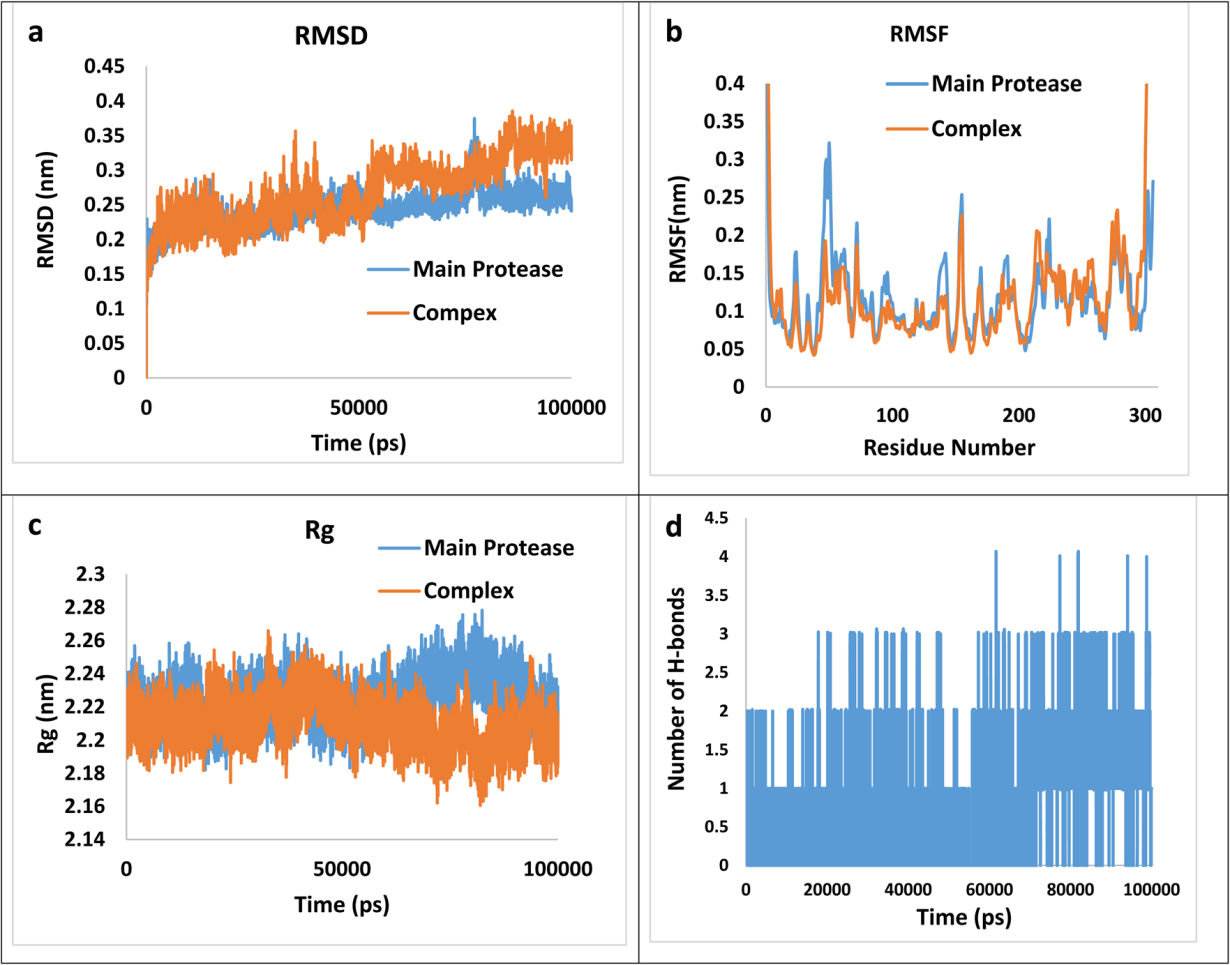
The RMSD (a), RMSF (b), *R*_g_ (c), and the numbers of hydrogen bonds plots (d) for the (4h)–main protease complex during 100 ns simulation.

Following equilibration, production MD simulations were conducted for 100 ns at a constant temperature of 300 K, with simulation trajectories saved every 1 fs. Each MD simulation was performed in triplicate for every complex, and the mean values of obtained parameters were used for structural analysis. The results of MD simulation were illustrated in Fig. 6(a–d) which a–d corresponds to the Root Mean Square Deviation (RMSD), the Root Mean Square Fluctuation (RMSF), the Radius of Gyration (*R*_g_) and the numbers of hydrogen bonds plots, respectively.

RMSD graphs illustrate the stability and conformational alterations observed in both the main protease and its associated complex with (4h) throughout the simulation. Mean RMSD values ranging from 0.1 to 0.3 nm are generally deemed satisfactory and indicative of stability. However, values exceeding 0.3 nm are considered unfavorable and are typically disregarded.^70^ The average calculated RMSD value stands at 0.27 nm, falling within an acceptable range. Furthermore, RMSF calculation proves valuable for identifying local fluctuations over the course of the simulation. The RMSF index provides insight into the flexibility of the protein chain relative to a reference frame. It measures the deviation of Cα atom coordinates from their average position, serving as a criterion for assessing these effects. The peaks observed in the RMSF plot indicate that certain residues exhibit greater fluctuations compared to others within the studied complex. Specifically, RMSF values for the complexes reveal significant fluctuations at the N- and C-termini in contrast to other regions. This phenomenon is commonly observed and does not require further investigation. Regarding the average values 0.117 and 0.120 nm for the main protease and the complex, respectively, can be concluded that the main protease does not involve abnormal fluctuations. The radius of gyration (*R*_g_) factor serves as a crucial metric for assessing the compactness of the ligand–protein complex throughout molecular simulations. It quantifies the extent of variation in distance between the center of mass of the protein atoms and its terminal. A lower *R*_g_ value in the ligand–protein complex indicates higher compactness, implying more effective interactions between the ligand and the protein. The mean *R*_g_ values for the complex and the main protease are 2.21 and 2.23 nm, suggesting more compactness of the complex. Finally, the number of hydrogen bonds during the simulation is an effective factor in the stability of the complex, the calculated average value of hydrogen bonds during the time of simulation is 0.81. Thus, besides the hydrogen bonding, other intermolecular forces may exert a greater effect on the total bending energy.

#### Binding energy studies

Binding energy analyses were conducted utilizing the molecular mechanics Poisson Boltzmann surface area (MM-PBSA) framework in GROMACS, which evaluates the nonbonded interaction energies within the complex. Furthermore, this methodology is employed to compute the binding free energy of enzyme complexes. Snapshots were collected at 1 ps intervals between 95 and 100 ns the simulations were utilized for post-processing binding free energies using the g_mmpbsa script.^71,72^ In general, the binding interaction of each ligand–enzyme complex is dissected into three main components: van der Waals contribution, electrostatic contribution, and solvation contribution. MMPBSA calculations reveal that the averaged binding free energy of the complex is −131.96 kJ mol^−1^. The values of van der Waals, electrostatic, polar solvation, and Solvent Accessible Surface Areas (SASA) energies are −173.97, −42.83, 100.96, and −16.12 kJ mol^−1^ respectively, showing that van der Waals and polar solvation energies are the most impactful factors in the calculated binding free energies.

## Conclusion

In summary, with the aim of discovering potent nonpeptidic and non-covalent inhibitors targeting 3CL^pro^, a key enzyme implicated in the replication of the coronavirus, an efficient two-step procedure was established for the synthesis of quinoxalino[2,1-*b*]quinazolin-12-one derivatives from synthetically accessible starting materials 2,3-dichloroquinoxaline and anthranilamides. Following that, molecular docking and molecular dynamics simulations were employed to assess the inhibitory potential of synthesized products against the main protease of COVID-19. According to molecular docking results, 6-(*p*-tolylamino)-12*H*-quinoxalino[2,1-*b*]quinazolin-12-one (4h) displayed the lowest binding affinity (−9.0 kcal mol^−1^), indicating their favorable orientation within the active site of the chymotrypsin-like cysteine protease (3CL^pro^). To gain deeper insights into ligand–protein interactions, molecular docking outcomes served as inputs for molecular dynamics simulations. The simulations revealed that the binding energy value of the (4h)–main protease complex is −131.96 kJ mol^−1^, also van der Waals and polar solvation energies are the most effective parameters on the total binding energy. Thus, the findings suggest that compound (4h) bearing *p*-tolylamino substitution on C-6 of quinoxalino[2,1-*b*]quinazolinone is a promising inhibitor against the main protease of COVID-19.

## Experimental

Melting points were measured by an Electrothermal type 9200 melting point apparatus. The ^1^H NMR (300 MHz) and the ^13^C NMR (75 MHz) spectra were obtained on a Bruker Avance DRX-300 Fourier transform spectrometer using tetramethylsilane as an internal standard. An Avatar 370 FT-IR Thermo Nicolet spectrometer was employed to record the IR spectra and a Varian Mat CH-7 instrument for scanning mass spectra at 70 eV. Micro analytical data were obtained on a Thermo Finnigan Flash EA 1112 microanalyzer.

### General procedure for the synthesis of 2-((3-chloroquinoxalin-2-yl)amino)-*N*-substituted benzamide derivatives (3a–h)

A mixture of appropriate 2-amino-*N*-substituted benzamide derivatives (1a–h) (1 mmol), 2,3-dichloroquinoxaline (2) (1 mmol, 0.199 g) and excess amount of KOH (6–8 granular pellets) in DMF (3 ml) was stirred at room temperature for 16 h. The progress of the reaction was monitored by TLC using chloroform : methanol 20 : 1 eluent. After the completion of the reaction, the mixture was cooled, poured into an ice/water bath and neutralized with aqueous 5% HCl solution. The resulting solid product was collected by filtration, washed with water (2 × 20 ml) and recrystallized from EtOH.

#### 2-((3-Chloroquinoxalin-2-yl)amino)-*N*-methylbenzamide (3a)

Yellow powder; yield 75%; m. p. 225–226 °C; IR (KBr disc, cm^−1^): *ν* 3295, 3215, 3060, 2937, 1633, 1592, 1530, 1451. ^1^H NMR (300 MHz, DMSO*-d*_6_): *δ*_H_ 2.87 (d, *J* = 4.5 Hz, 3H, NCH_3_), 7.13–7.18 (m, 1H, Ar-H), 7.49–7.61 (m, 2H, 2Ar-H), 7.65–7.70 (m, 1H, Ar-H), 7.75–7.84 (m, 3H, 3Ar-H), 8.82 (br s, 1H, NH), 9.09 (dd, *J* = 8.4 Hz, *J* = 0.7 Hz, 1H, Ar-H), 12.32 (s, 1H, NH̲–C=O). ^13^C NMR (75 MHz, DMSO-*d*_6_, ppm): *δ*_C_ 26.9, 120.0, 120.4, 122.3, 126.7, 126.8, 127.9, 128.5, 130.9, 132.4, 136.8, 139.2, 139.9, 140.4, 145.4, 169.5. MS (*m*/*z*) = 312 (M^+^). Anal. calcd for C_16_H_13_ClN_4_O (%): C, 61.45; H, 4.19; N, 17.91. Found: C, 61.43; H, 4.14; N, 17.80.

#### 2-((3-Chloroquinoxalin-2-yl)amino)-*N*-ethylbenzamide (3b)

Yellow powder; yield 64%; m. p. 207–208 °C; IR (KBr disc, cm^−1^): *ν* 3329, 3256, 3215, 3113, 3056, 2970, 2933, 2876, 1635, 1591, 1529, 1056. ^1^H NMR (300 MHz, DMSO-*d*_6_): *δ*_H_ 1.19 (t, *J* = 7.2 Hz, 3H, CH_3_), 3.33–3.41 (m, 2H, NCH_2_), 7.15 (m, 1H, Ar-H), 7.46–7.52 (m, 1H, Ar-H), 7.55–7.68 (m, 2H, 2Ar-H), 7.73–7.85 (m, 3H, 3Ar-H), 8.83 (br s, 1H, NH), 9.07 (d, *J* = 8.1 Hz, 1H, Ar-H), 12.27 (s, 1H, NH̲–C=O). ^13^C NMR (75 MHz, DMSO-*d*_6_, ppm): *δ*_C_ 15.0, 34.6, 120.0, 120.7, 122.2, 126.6, 126.7, 127.9, 128.6, 130.8, 132.3, 136.8, 139.1, 139.9, 140.4, 145.4, 168.8; MS (*m*/*z*) = 326 (M^+^). Anal. calcd for C_17_H_15_ClN_4_O (%): C, 62.48; H, 4.63; N, 17.15. Found: C, 62.43; H, 4.60; N, 17.13.

#### 2-((3-Chloroquinoxalin-2-yl)amino)-*N*-propylbenzamide (3c)

Yellow powder; yield 69%; m. p. 216–217 °C; IR (KBr disc, cm^−1^): *ν* 3321, 3056, 3031, 2961, 2933, 2872, 1646, 1592, 1524, 1450, 1060. ^1^H NMR (300 MHz, DMSO-*d*_6_): *δ*_H_ 0.94 (t, *J* = 7.4 Hz, 3H, CH_3_), 1.54–1.66 (m, 2H, CH_2_), 3.26–3.33 (m, 2H, NCH_2_), 7.15–7.20 (m, 1H, Ar-H), 7.52–7.63 (m, 2H, 2Ar-H), 7.68–7.73 (m, 1H, Ar-H), 7.79–7.86 (m, 3H, 3Ar-H), 8.84 (br s, 1H, NH), 9.09 (d, *J* = 7.7 Hz, 1H, Ar-H), 12.21 (s, 1H, NH̲–C=O). ^13^C NMR (75 MHz, DMSO-*d*_6_, ppm): *δ*_C_ 11.9, 22.6, 41.5, 120.0, 120.9, 122.3, 126.7, 126.8, 127.9, 128.7, 131.0, 132.3, 136.8, 139.2, 139.9, 140.3, 145.5, 169.0. MS (*m*/*z*) = 340 (M^+^). Anal. calcd for C_18_H_17_ClN_4_O (%): C, 63.44; H, 5.03; N, 16.44. Found: C, 63.40; H, 5.01; N, 16.42.

#### 2-((3-Chloroquinoxalin-2-yl)amino)-*N*-isopropylbenzamide (3d)

Yellow powder; yield 64%; m. p. 230–231 °C; IR (KBr disc, cm^−1^): *ν* 3333, 3243, 3206, 3116, 2969, 2871, 1632, 1591, 1524, 1450, 1056. ^1^H NMR (300 MHz, DMSO-*d*_6_): *δ*_H_ 1.22 (d, *J* = 6.5 Hz, 6H, 2CH_3_), 4.14–4.25 (m, 1H, NCH_2_), 7.18 (m, 1H, Ar-H), 7.54–7.64 (m, 2H, 2Ar-H), 7.70–7.75 (m, 1H, Ar-H), 7.81–7.87 (m, 3H, 3Ar-H), 8.64 (br s, 1H, NH), 9.08 (d, *J* = 8.3 Hz, 1H, Ar-H), 12.18 (s, 1H, NH̲–C=O). ^13^C NMR (75 MHz, DMSO-*d*_6_, ppm): *δ*_C_ 22.6, 41.6, 120.0, 121.1, 122.3, 126.7, 126.8, 128.0, 128.9, 131.0, 132.3, 136.8, 139.2, 140.0, 140.2, 145.5, 168.2. MS (*m*/*z*) = 340 (M^+^). Anal. calcd for C_18_H_17_ClN_4_O (%): C, 63.44; H, 5.03; N, 16.44. Found: C, 63.42; H, 5.02; N, 16.42.

#### *N*-Butyl-2-((3-chloroquinoxalin-2-yl)amino)benzamide (3e)

Yellow powder; yield 57%; m. p. 180–181 °C; IR (KBr disc, cm^−1^): *ν* 3316, 3248, 3211, 3109, 3056, 2962, 2928, 2859, 1634, 1593, 1529, 1450, 1057. ^1^H NMR (300 MHz, DMSO-*d*_6_): *δ*_H_ 0.93 (t, *J* = 7.3 Hz, 3H, CH_3_), 1.31–1.43 (m, 2H, CH_2_), 1.52–1.61 (m, 2H, CH_2_), 3.30–3.43 (m, 2H, NCH_2_), 7.16–7.21 (m, 1H, Ar-H), 7.55–7.64 (m, 2H, 2Ar-H), 7.71–7.76 (m, 1H, Ar-H), 7.82–7.87 (m, 3H, 3Ar-H), 8.83 (br s, 1H, NH), 9.09 (d, *J* = 8.4 Hz, 1H, Ar-H), 12.20 (s, 1H, NH̲–C=O). ^13^C NMR (75 MHz, DMSO-*d*_6_, ppm): *δ*_C_ 14.2, 20.1, 31.5, 39.4, 120.0, 121.0, 122.4, 126.7, 126.9, 128.0, 128.7, 131.1, 132.4, 136.9, 139.2, 140.0, 140.2, 145.5, 169.0. MS (*m*/*z*) = 354 (M^+^). Anal. calcd for C_19_H_19_ClN_4_O (%): C, 64.31; H, 5.40; N, 15.79. Found: C, 64.28; H, 5.38; N, 15.75.

#### 2-((3-Chloroquinoxalin-2-yl)amino)-*N*-isobutylbenzamide (3f)

Yellow powder; yield 58%; m. p 186–187 °C; IR (KBr disc, cm^−1^): *ν* 3320, 3252, 3215, 3056, 2955, 2921, 2868, 1634, 1592, 1529, 1448, 1305, 1056. ^1^H NMR (300 MHz, DMSO-*d*_6_): *δ*_H_ 0.93 (d, *J* = 6.6 Hz, 6H, 2CH_3_), 1.85–1.96 (m, *J* = 6.7 Hz, 1H, CH), 3.17 (t, *J* = 6.4 Hz, 2H, NCH_2_), 7.17 (t, *J* = 7.4 Hz, 1H, Ar-H), 7.50–7.63 (m, 2H, 2Ar-H), 7.66–7.71 (m, 1H, Ar-H), 7.77–7.86 (m, 3H, 3Ar-H), 8.85 (br s, 1H, NH), 9.06 (d, *J* = 8.3 Hz, 1H, Ar-H), 12.11 (s, 1H, NH̲–C=O). ^13^C NMR (75 MHz, DMSO-*d*_6_, ppm): *δ*_C_ 20.7, 28.5, 47.1, 120.0, 121.1, 122.3, 126.7, 126.8, 128.0, 128.7, 131.0, 132.3, 136.8, 139.1, 139.9, 140.2, 145.4, 169.1. MS (*m*/*z*) = 354 (M^+^). Anal. calcd for C_19_H_19_ClN_4_O (%): C, 64.31; H, 5.40; N, 15.79. Found: C, 64.28; H, 5.38; N, 15.77.

#### *N*-Benzyl-2-((3-chloroquinoxalin-2-yl)amino)benzamide (3g)

Yellow powder; yield 49%; m. p.170–171 °C; IR (KBr disc, cm^−1^): *ν* 3471, 3362, 3301, 3060, 3027, 2921, 2851, 1667, 1631, 1591, 1528, 1451, 1054. ^1^H NMR (300 MHz, DMSO-*d*_6_): *δ*_H_ 4.57 (d, *J* = 5.9 Hz, 2H, NCH_2_), 7.17–7.42 (m, 5H, 5Ar-H_Ph_), 7.52–7. 58 (m, 1H, Ar-H), 7.60–7.68 (m, 2H, 2Ar-H), 7.70–7.74 (m, 1H, Ar-H), 7.79–7.85 (m, *J* = 8.3 Hz, *J* = 0.9 Hz, 2H, 2Ar-H), 7.93 (dd, *J* = 7.9 Hz, *J* = 1.1 Hz, 1H, Ar-H), 9.11 (d, *J* = 7.8 Hz, 1H, Ar-H), 9.43 (br s, 1H, NH), 12.18 (s, 1H, NH̲–C=O). ^13^C NMR (75 MHz, DMSO-*d*_6_, ppm): *δ*_C_ 43.1, 120.1, 120.5, 122.4, 126.7, 126.9, 127.4, 127.7, 128.0, 128.7, 128.8, 131.0, 132.6, 136.9, 139.2, 139.6, 139.9, 140.4, 145.4, 169.1. MS (*m*/*z*) = 388 (M^+^). Anal. calcd for C_22_H_17_ClN_4_O (%): C, 67.95; H, 4.41; N, 14.41. Found: C, 67.91; H, 4.40; N, 14.39.

#### 2-((3-Chloroquinoxalin-2-yl)amino)-*N*-(*p*-tolyl)benzamide (3h)

Yellow powder; yield 55%; m. p.173–174 °C; IR (KBr disc, cm^−1^): *ν* 3466, 3362, 3276, 3186, 3105, 3029, 2917, 1658, 1635, 1598, 1514, 1450, 1320, 1255. MS (*m*/*z*) = 388 (M^+^). Anal. calcd for C_22_H_16_N_4_O (%): C, 67.95; H, 4.41; N, 14.41. Found: C, 67.94; H, 4.38; N, 14.39.

### General procedure for the synthesis of (4a–h)

A mixture of the appropriate 2-((3-chloroquinoxalin-2-yl)amino)-*N*-substituted benzamide derivatives (3a–h) (1 mmol) and K_2_CO_3_ (4 mmol, 0.552 g) in DMF (3 ml) was heated at 125 °C for 5 h. After the completion of the reaction (monitored by TLC, CHCl_3_ : MeOH, 20 : 1), the mixture was cooled, poured onto ice water and neutralized with aqueous 5% HCl solution. The resulting solid product was collected by filtration and recrystallized from acetone to form crystalline yellow needle precipitates.

#### 6-(Methylamino)-12*H*-quinoxalino[2,1-*b*]quinazolin-12-one (4a)

Yellow needle crystal; yield 75%; m. p. 168–169 °C; IR (KBr disc, cm^−1^): *ν* 3417, 3137, 3064, 3002, 2925, 2892, 1695, 1623, 1585, 1413, 1200. ^1^H NMR (300 MHz, CDCl_3_): *δ*_H_ 3.21 (d, *J* = 5.1 Hz, 3H, NCH_3_), 7.12 (br s, 1H, NH, D_2_O exchangeable), 7.23–7.30 (m, 1H, Ar-H), 7.38–7.44 (m, 1H, Ar-H), 7.51–7.62 (m, 2H, 2Ar-H),7.68–7.81 (m, 2H, 2Ar-H), 8.41 (dd, *J* = 8.1 Hz, *J* = 0.9 Hz, 1H, Ar-H), 9.42 (dd, *J* = 8.7 Hz, *J* = 1.1 Hz, 1H, Ar-H). ^13^C NMR (75 MHz, CDCl_3_): *δ*_C_ 28.0, 120.4, 121.5, 123.5, 125.2, 126.3, 127.0, 127.4, 127.5, 134.6, 135.8, 136.5, 144.9, 149.9, 161.8. MS (*m*/*z*) = 276 (M^+^). Anal. calcd for C_16_H_12_N_4_O (%): C, 69.55; H, 4.38; N, 20.28. Found: C, 69.53; H, 4.37; N, 20.26.

#### 6-(Ethylamino)-12*H*-quinoxalino[2,1-*b*]quinazolin-12-one (4b)

Yellow needle crystal; yield 82%; m. p. 138–139 °C; IR (KBr disc, cm^−1^): *ν* 3394, 3133, 3064, 2962, 2868, 1698, 1623, 1583, 1556, 1455, 1315. ^1^H NMR (300 MHz, CDCl_3_): *δ*_H_ 1.44 (t, *J* = 7.2 Hz, 3H, CH_3_), 3.71–3.79 (m, 2H, NCH_2_), 7.19 (br s, 1H, NH), 7.27–7.33 (m, 1H, Ar-H), 7.43–7.48 (m, 1H, Ar-H), 7.59–7.65 (m, 2H, 2Ar-H), 7.82–7.90 (m, 2H, 2Ar-H), 8.50 (d, *J* = 8.1 Hz, 1H, Ar-H), 9.46 (dd, *J* = 8.7 Hz, *J* = 1.2 Hz, 1H, Ar-H). ^13^C NMR (75 MHz, CDCl_3_): *δ*_C_ 14.7, 36.0, 120.4, 121.6, 123.5, 125.2, 126.4, 127.2, 127.4, 127.6, 127.7, 134.7, 135.9, 136.6, 145.1, 149.3, 162.0. MS (*m*/*z*) = 290 (M^+^). Anal. calcd for C_17_H_14_N_4_O (%): C, 70.33; H, 4.86; N, 19.30. Found: C, 70.30; H, 4.84; N, 19.28.

#### 6-(Propylamino)-12*H*-quinoxalino[2,1-*b*]quinazolin-12-one (4c)

Yellow needle crystal; yield 87%; m. p 150–151 °C; IR (KBr disc, cm^−1^): *ν* 3389, 3145, 3060, 3007, 2959, 2931, 2859,1700, 1623, 1585, 1526, 1194. ^1^H NMR (300 MHz, CDCl_3_): *δ*_H_1.12 (t, *J* = 7.4 Hz, 3H, CH_3_), 1.81–1.88 (m, *J* = 7.3 Hz, 2H, CH_2_), 3.66 (q, *J* = 6.7 Hz, 2H, NCH_2_), 7.23 (br s, 1H, NH), 7.25–7.32 (m, 1H, Ar-H), 7.42–7.47 (m, 1H, Ar-H), 7.58–7.64 (m, 2H, 2Ar-H), 7.80–7.89 (m, 2H, 2Ar-H), 8.48 (d, *J* = 8.1 Hz, 1H, Ar-H), 9.46 (d, *J* = 8.6 Hz, 1H, Ar-H). ^13^C NMR (75 MHz, CDCl_3_): *δ*_C_ 11.7, 22.6, 43.0, 120.4, 121.5, 123.5, 125.1, 126.3, 127.2, 127.4, 127.6, 127.7, 134.7, 135.9, 136.6, 145.0, 149.4, 162.0. MS (*m*/*z*) = 304 (M^+^). Anal. calcd for C_18_H_16_N_4_O (%): C, 71.04; H, 5.30; N, 18.41. Found: C, 71.01; H, 5.28; N, 18.39.

#### 6-(Isopropylamino)-12*H*-quinoxalino[2,1-*b*]quinazolin-12-one (4d)

Yellow needle crystal; yield 70%; m. p. 153–154 °C; IR (KBr disc, cm^−1^): *ν* 3391, 3133, 3064, 2968, 2925, 2868, 1698, 1623, 1583, 1555, 1522, 1198. ^1^H NMR (300 MHz, CDCl_3_): *δ*_H_ 1.44 (d, *J* = 6.5 Hz, 6H, 2CH_3_), 4.46–4.58 (m, 1H, NCH_2_), 7.10 (br s, 1H, NH), 7.27–7.33 (m, 1H, Ar-H), 7.43–7.48 (m, 1H, Ar-H), 7.60–7.65 (m, 2H, 2Ar-H), 7.85–7.92 (m, 2H, 2Ar-H), 8.51 (d, *J* = 8.1 Hz, 1H, Ar-H), 9.46 (dd, *J* = 8.7 Hz, *J* = 1.2 Hz, 1H, Ar-H). ^13^C NMR (75 MHz, CDCl_3_): *δ*_C_ 22.7, 42.7, 119.0, 120.4, 121.6, 123.4, 125.1, 126.4, 127.2, 127.4, 127.5, 127.6, 134.7, 136.7, 145.3, 148.8, 162.3. MS (*m*/*z*) = 304 (M^+^). Anal. calcd for C_18_H_16_N_4_O (%): C, 71.04; H, 5.30; N, 18.41. Found: C, 71.03; H, 5.27; N, 18.38.

#### 6-(Butylamino)-12*H*-quinoxalino[2,1-*b*]quinazolin-12-one (4e)

Yellow needle crystal; yield 92%; m. p. 121–122 °C; IR (KBr disc, cm^−1^): *ν* 3382, 3141, 3060, 2956, 2932, 2868, 1699, 1624, 1586, 1558, 1357, 1194. ^1^H NMR (300 MHz, CDCl_3_): *δ*_H_ 1.06 (t, *J* = 7.3 Hz, 3H, CH_3_), 1.50–1.62 (m, 2H, CH_2_), 1.76–1.85 (m, 2H, CH_2_), 3.69 (q, *J* = 6.6 Hz, 2H, NCH_2_), 7.19 (br s, 1H, NH), 7.25–7.31 (m, 1H, Ar-H), 7.41–7.46 (m, 1H, Ar-H), 7.56–7.63 (m, 2H, 2Ar-H), 7.78–7.87 (m, 2H, 2Ar-H), 8.47 (dd, *J* = 8.2 Hz, *J* = 0.8 Hz, 1H, Ar-H), 9.45 (dd, *J* = 8.7 Hz, *J* = 1.2 Hz, 1H, Ar-H). ^13^C NMR (75 MHz, CDCl_3_): *δ*_C_ 14.0, 20.4, 31.5, 40.9, 120.4, 121.5, 123.4, 125.1, 126.3, 127.1, 127.4, 127.5, 127.6, 134.7, 135.9, 136.6, 145.0, 149.4, 162.0. MS (*m*/*z*) = 318 (M^+^). Anal. calcd for C_19_H_18_N_4_O (%): C, 71.68; H, 5.70; N, 17.60. Found: C, 71.65; H, 5.69; N, 17.57.

Single crystal (thin needle, 0.055 × 0.092 × 0.174 mm) of compound (4e) was grown by slow evaporation of ethanol solution. Diffraction (crystal was cooled to 100 K) to 1.5 Å resolution with reasonable statistic (*R*-factor 0.4) allowed to determine approximate unit cell (*a* = 7.01, *b* = 10.24, *c* = 20.92, *α* = 90.11, *β* = 90.03, *γ* = 90.15) and solve the structure in *P*1 space group (*Z* = 4), as weak reflections did not allow to refine unit cell and assign correct space group. Nevertheless, atoms connectivity confirmed the structure of compound (4e). Then it was subjected to slow evaporation from aqueous perchloric acid–ethanol mixture, which resulted in large crystals of (4e)–perchlorate salt, suitable for high resolution X-ray diffraction experiment. Full crystallographic report is available in ESI† (CCDC deposition number 2357348).

#### 6-(Isobutylamino)-12*H*-quinoxalino[2,1-*b*]quinazolin-12-one (4f)

Yellow needle crystal; yield 85%; m. p. 124–125 °C; IR (KBr disc, cm^−1^): *ν* 3396, 3150, 3060, 2958, 2917, 2868, 1685, 1623, 1574, 1556, 1312, 1196. ^1^H NMR (300 MHz, CDCl_3_): *δ*_H_ 1.12 (d, *J* = 6.7 Hz, 6H, 2CH_3_), 2.07–2.21 (m, 1H, CH), 3.53 (t, *J* = 6.4 Hz, 2H, NCH_2_), 7.25 (br s, 1H, NH), 7.28–7.31 (m, 1H, Ar-H), 7.41–7.47 (m, 1H, Ar-H), 7.57–7.63 (m, 2H, 2Ar-H), 7.80–7.88 (m, 2H, 2Ar-H), 8.48 (d, *J* = 8.1 Hz, 1H, Ar-H), 9.45 (dd, *J* = 8.6 Hz, *J* = 1.1 Hz, 1H, Ar-H). ^13^C NMR (75 MHz, CDCl_3_): *δ*_C_ 20.5, 28.3, 48.6, 120.4, 121.5, 123.4, 125.1, 126.3, 127.2, 127.4, 127.5, 127.6, 134.7, 135.9, 136.6, 145.0, 149.5, 162.0. MS (*m*/*z*) = 318 (M^+^). Anal. calcd for C_19_H_18_N_4_O (%): C, 71.68; H, 5.70; N, 17.60. Found: C, 71.67; H, 5.68; N, 17.57.

#### 6-(Benzylamino)-12*H*-quinoxalino[2,1-*b*]quinazolin-12-one (4g)

Yellow needle crystal; yield 74%; m. p. 160–161 °C; IR (KBr disc, cm^−1^): *ν* 3403, 3137, 3064, 3023, 2864, 1697, 1623, 1574, 1556, 1454, 1369, 1313 ^1^H NMR (300 MHz, CDCl_3_): *δ*_H_ 4.91 (d, *J* = 5.8 Hz, 2H, NCH_2_), 7.29–7.46 (m, 5H, 5Ar-H_Ph_), 7.50–7.55 (m, 3H, (2Ar-H, NH)), 7.58–7.68 (m, 2H, 2Ar-H), 7.77–7.87 (m, 2H, 2Ar-H), 8.49 (d, *J* = 8.1 Hz, 1H, Ar-H), 9.48 (d, *J* = 8.5 Hz, 1H, Ar-H). ^13^C NMR (75 MHz, CDCl_3_): *δ*_C_ 45.2, 120.4, 121.6, 123.8, 125.4, 126.5, 127.2, 127.4, 127.5, 127.6, 127.7, 128.1, 128.7, 134.7, 135.8, 136.4, 138.6, 145.0, 149.3, 162.0. MS (*m*/*z*) = 352 (M^+^). Anal. calcd for C_22_H_16_N_4_O (%): C, 74.98; H, 4.58; N, 15.90. Found: C, 74.97; H, 4.55; N, 15.88.

#### 6-(*p*-Tolylamino)-12*H*-quinoxalino[2,1-*b*]quinazolin-12-one (4h)

Yellow needle crystal; yield 72%; m. p. 165–166 °C; IR (KBr disc, cm^−1^): *ν* 3346, 3031, 2921, 2847, 1701, 1626, 1585, 1535, 1459, 1343, 1315, 1176. ^1^H NMR (300 MHz, CDCl_3_): *δ*_H_ 2.34 (s, 3H, CH_3_), 7.24–7.27 (m, 2H, 2Ar-H), 7.34–7.40 (m, 1H, Ar-H), 7.48–7.53 (m, 1H, Ar-H), 7.62–7.66 (m, 1H, Ar-H), 7.70–7.75 (m, 1H, Ar-H), 8.02–8.06 (m, 4H, 4Ar-H_Ph_), 8.40 (d, *J* = 7.8 Hz, 1H, Ar-H), 9.42 (d, *J* = 8.6 Hz, 1H, Ar-H), 9.77 (s, 1H, NH). ^13^C NMR (75 MHz, DMSO-*d*_6_, ppm): *δ*_C_ 23.2, 122.7, 122.9, 123.8, 126.8, 128.3, 129.2, 129.7, 129.9, 130.6, 131.8, 134.7, 137.8, 138.1, 138.6, 139.5, 147.1, 149.5, 164.3. MS (*m*/*z*) = 352 (M^+^). Anal. calcd for C_22_H_16_N_4_O (%): C, 74.98; H, 4.58; N, 15.90. Found: C, 74.95; H, 4.57; N, 15.87.

## Data availability

Crystallographic data for compound (4e) has been deposited at the Cambridge Crystallographic Data Centre under deposition no. CCDC 2357348. The other data sets used and analyzed during the current study are available in ESI.†

## Conflicts of interest

The authors declares that they have no conflicts of interest.

## Supplementary Material

RA-014-D4RA06025C-s001

RA-014-D4RA06025C-s002
