# Supplementary material for: Design and synthesis of novel main protease inhibitors of COVID-19: quinoxalino[2,1-b]quinazolin-12-ones
Source: RSC Adv. 2024 Sep 13;14(40):29122–33. doi: 10.1039/d4ra06025c (PMC11393744; doi:10.1039/d4ra06025c)
Supplement: RA-014-D4RA06025C-s001 [file RA-014-D4RA06025C-s001.pdf]

Supplementary Information (SI) for RSC Advances.
This journal is © The Royal Society of Chemistry 2024

Supporting Information

Design and Synthesis of Novel Main Protease Inhibitors of COVID-19: Quinoxalino[2,1-*b*]quinazolin-12-ones

Atefeh Tirehdast[1], Seddigheh Sheikhi-Mohammareh[1], Hossein Sabet-Sarvestani[1,2], Michael G. Organ,[3] Volodymyr Semeniuchenko[3], Ali Shiri[1]*

[1]Department of Chemistry, Faculty of Science, Ferdowsi University of Mashhad, Mashhad, Iran.

[2]Department of Food Additives, Food Science and Technology Research Institute, Research Center for Iranian Academic Center for Education, Culture and Research (ACECR), Khorasan Razavi Branch, Mashhad, Iran.

[3]Department of Chemistry and Biomolecular Sciences, Faculty of Science, University of Ottawa, Ottawa, Canada.

[*]Corresponding Author E-mail: alishiri@um.ac.ir

Table of Contents Page

1. ^{1}H, and ^{13}C NMR spectra of compounds **(3a-g), (4a-h)**……………………….......S2-31
2. D_2O-exchangeable spectrum of compound **(4a)**……………..……..…......................S32
3. COSY spectrum of compound **(4a)** ………………………..……...........................S33
4. NOESY spectrum of compound **(4a)**…….………… …………..……..................S34
5. Crystallographic data....………………………...S35-S37

^{1}H NMR spectrum of compound **(3a)**

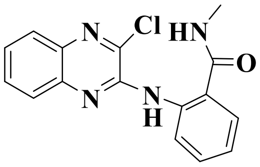

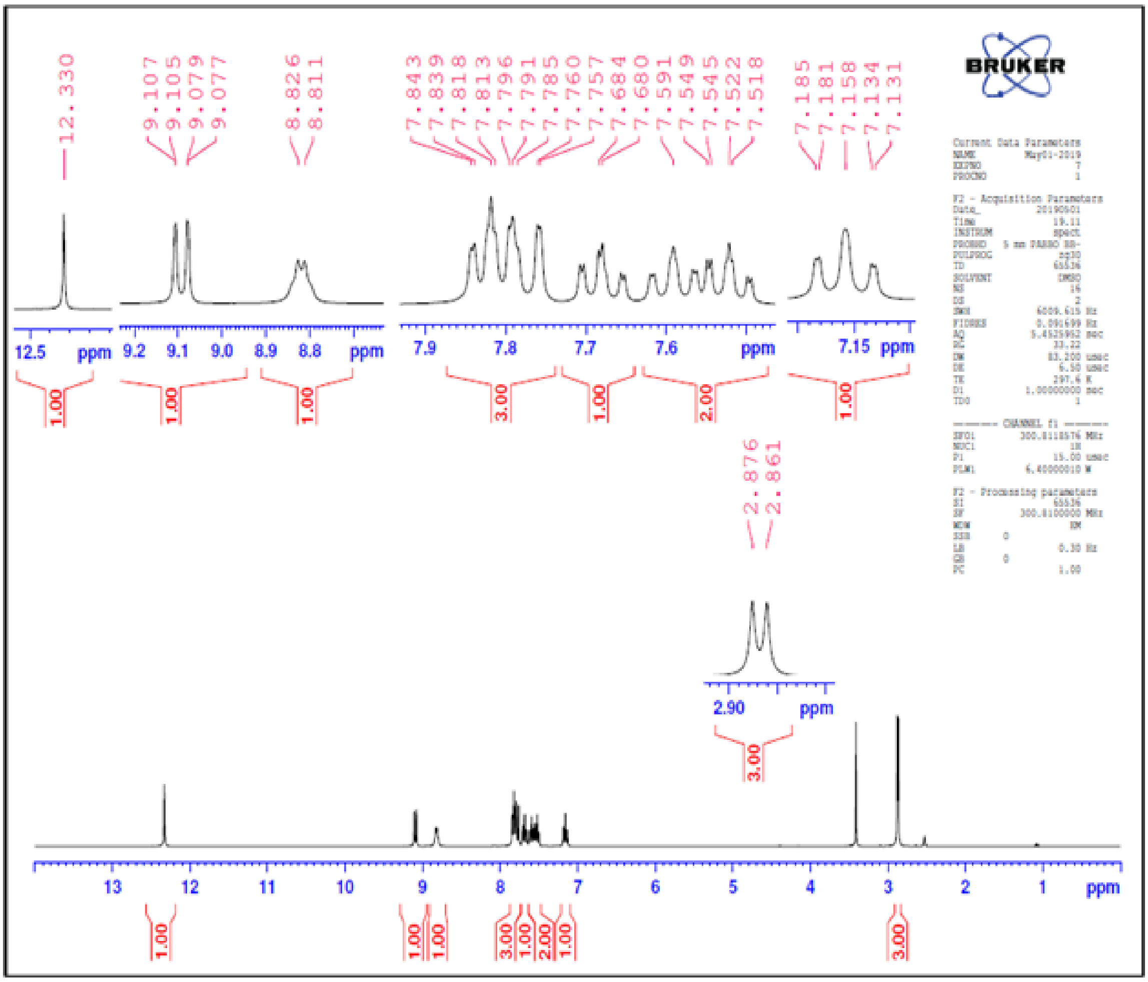

^{13}C NMR spectrum of compound **(3a)**

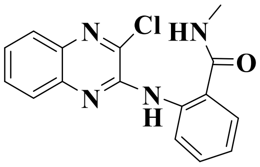

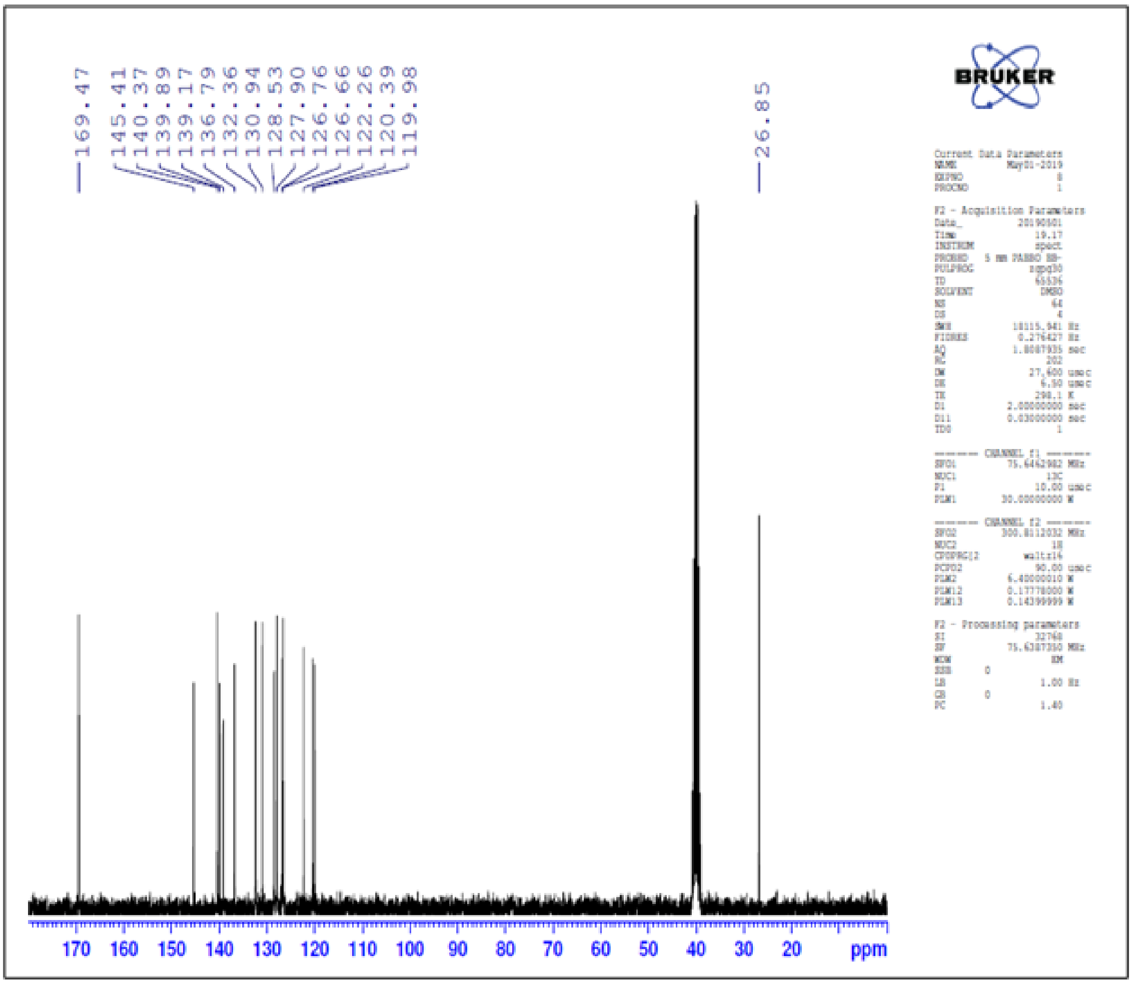

^{1}H NMR spectrum of compound **(3b)**

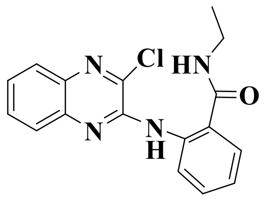

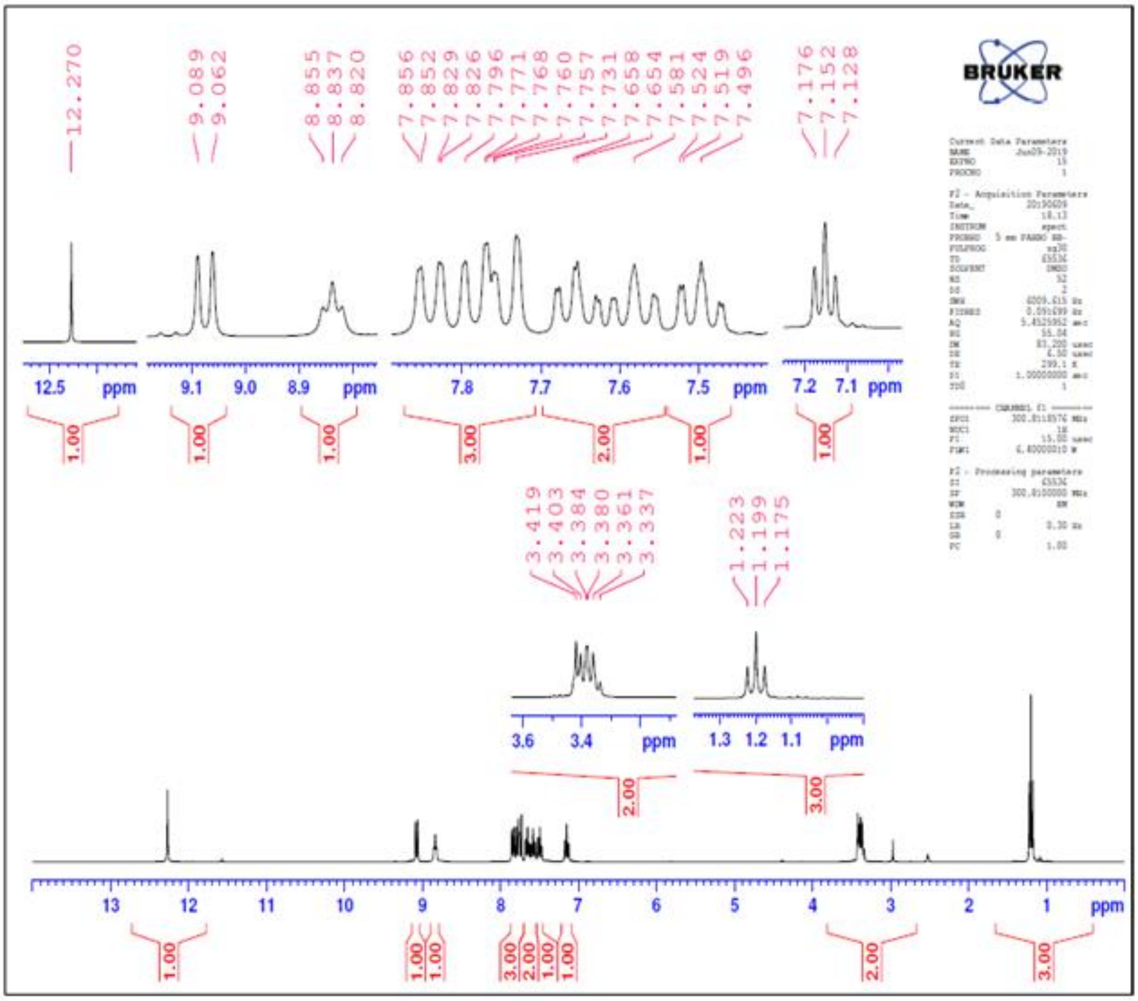

^{13}C NMR spectrum of compound **(3b)**

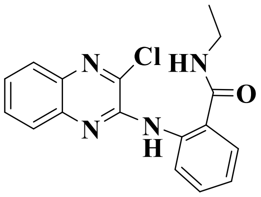

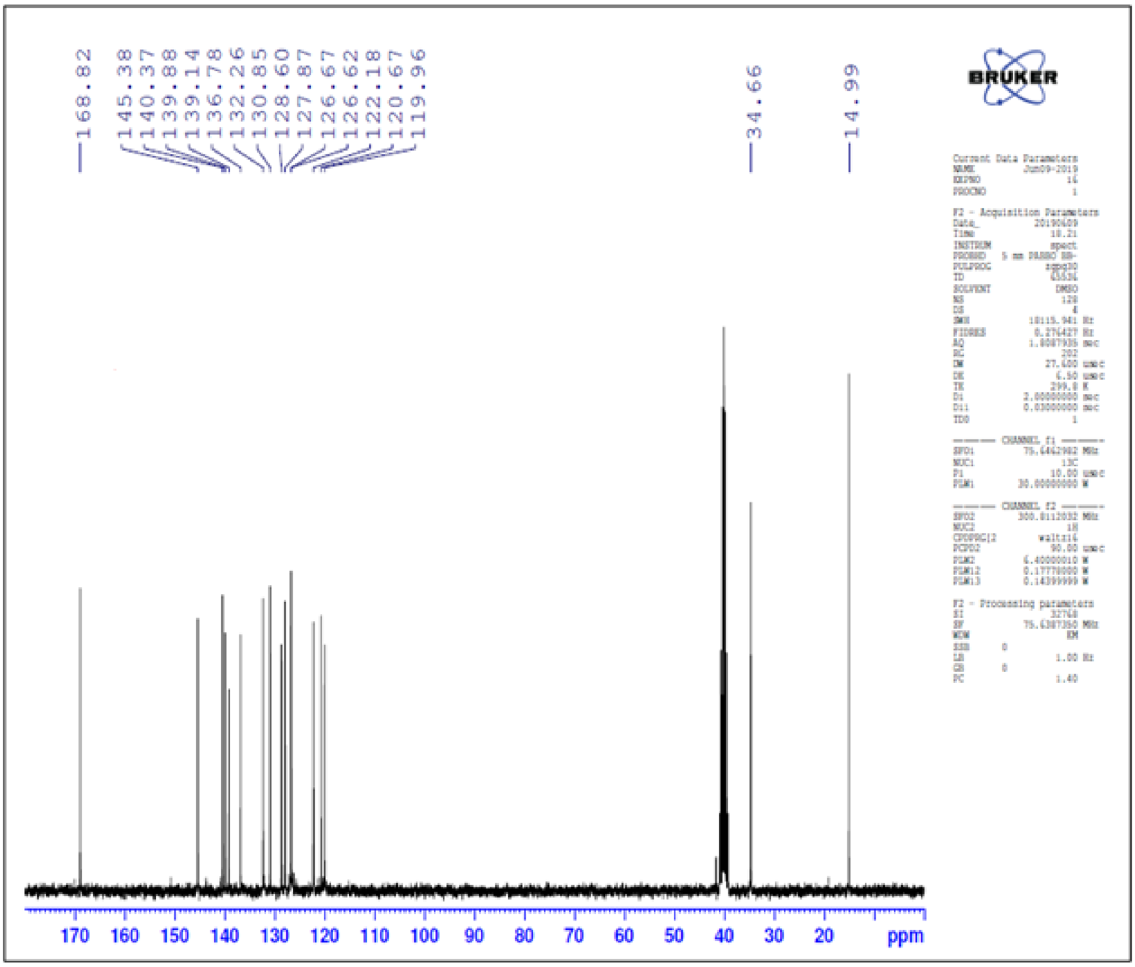

^{1}H NMR spectrum of compound **(3c)**

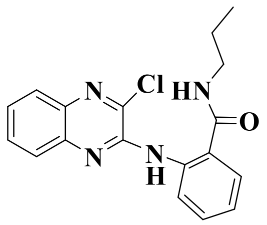

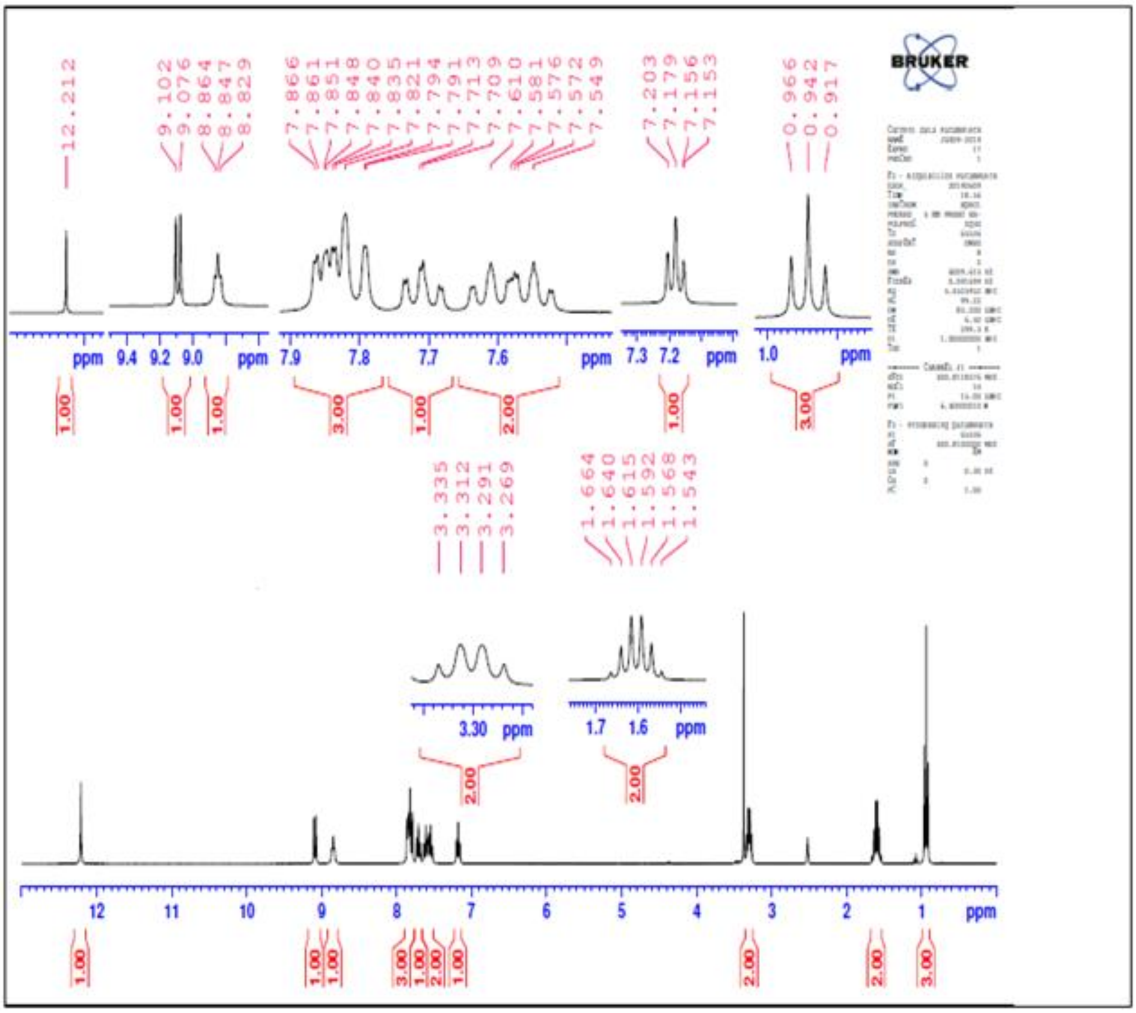

^{13}C NMR spectrum of compound **(3c)**

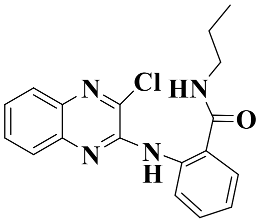

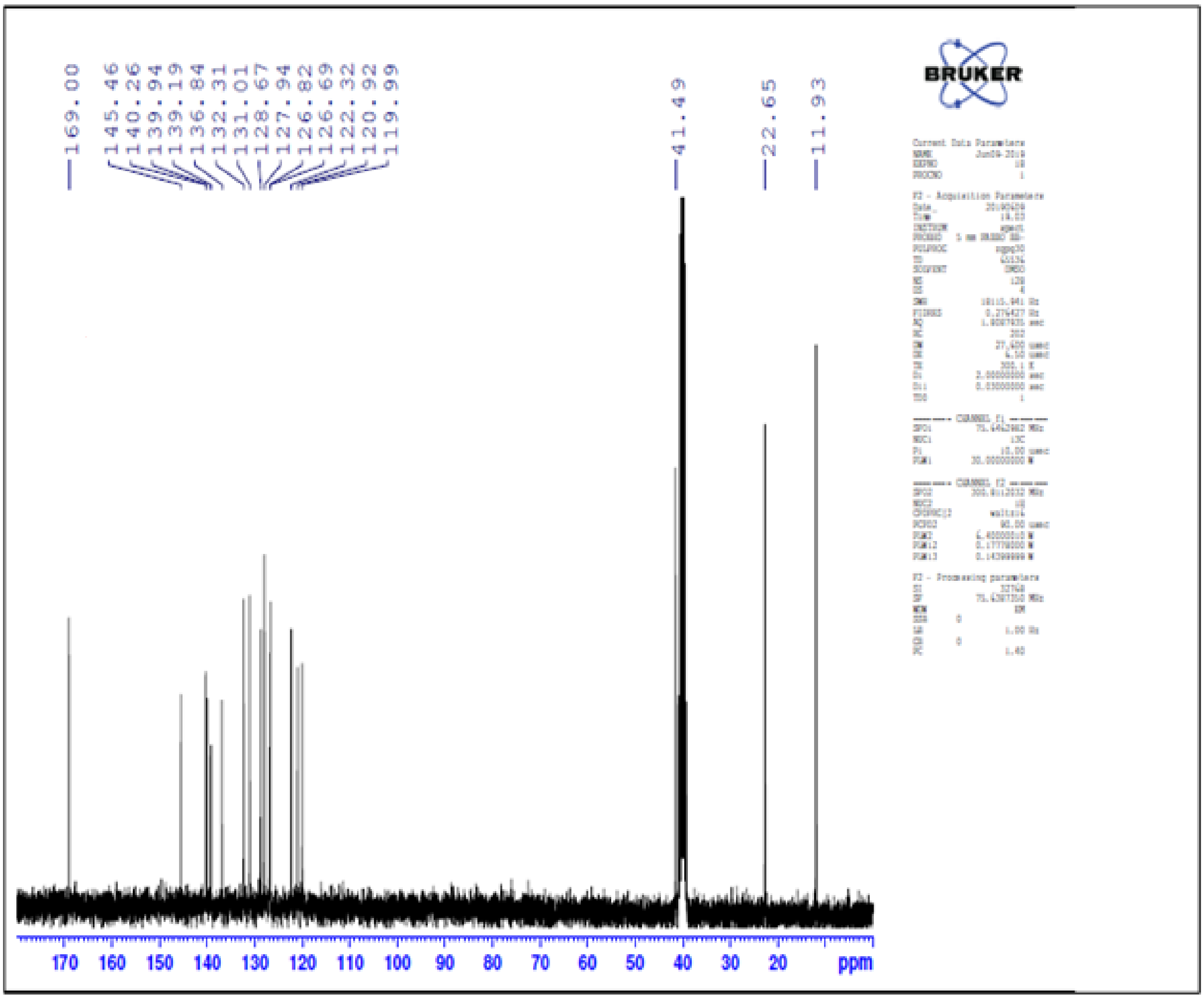

^{1}H NMR spectrum of compound **(3d)**

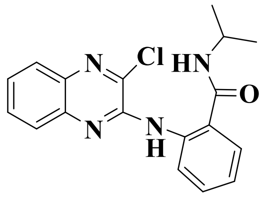

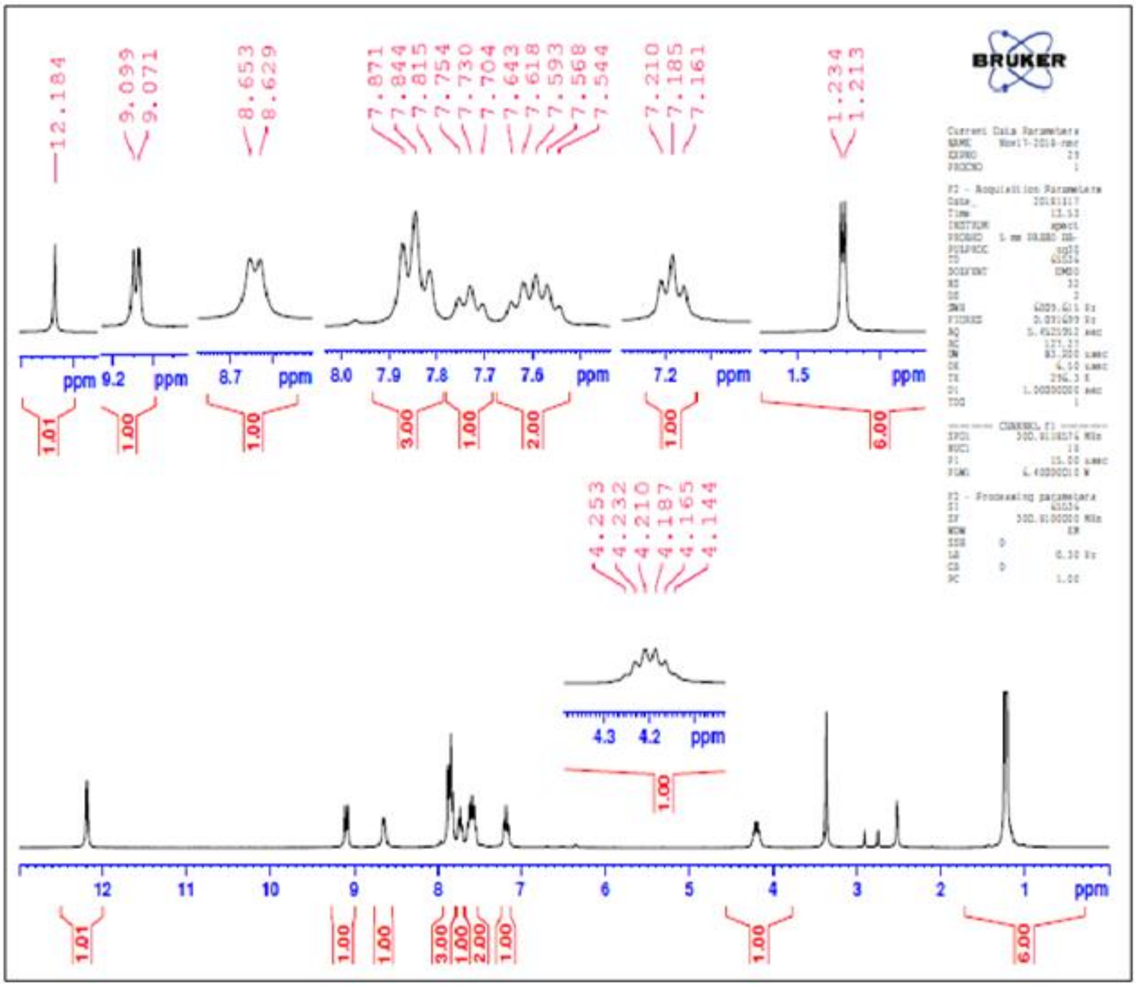

^{13}C NMR spectrum of compound **(3d)**

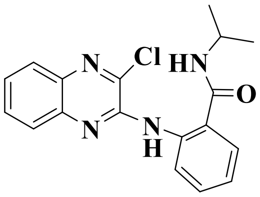

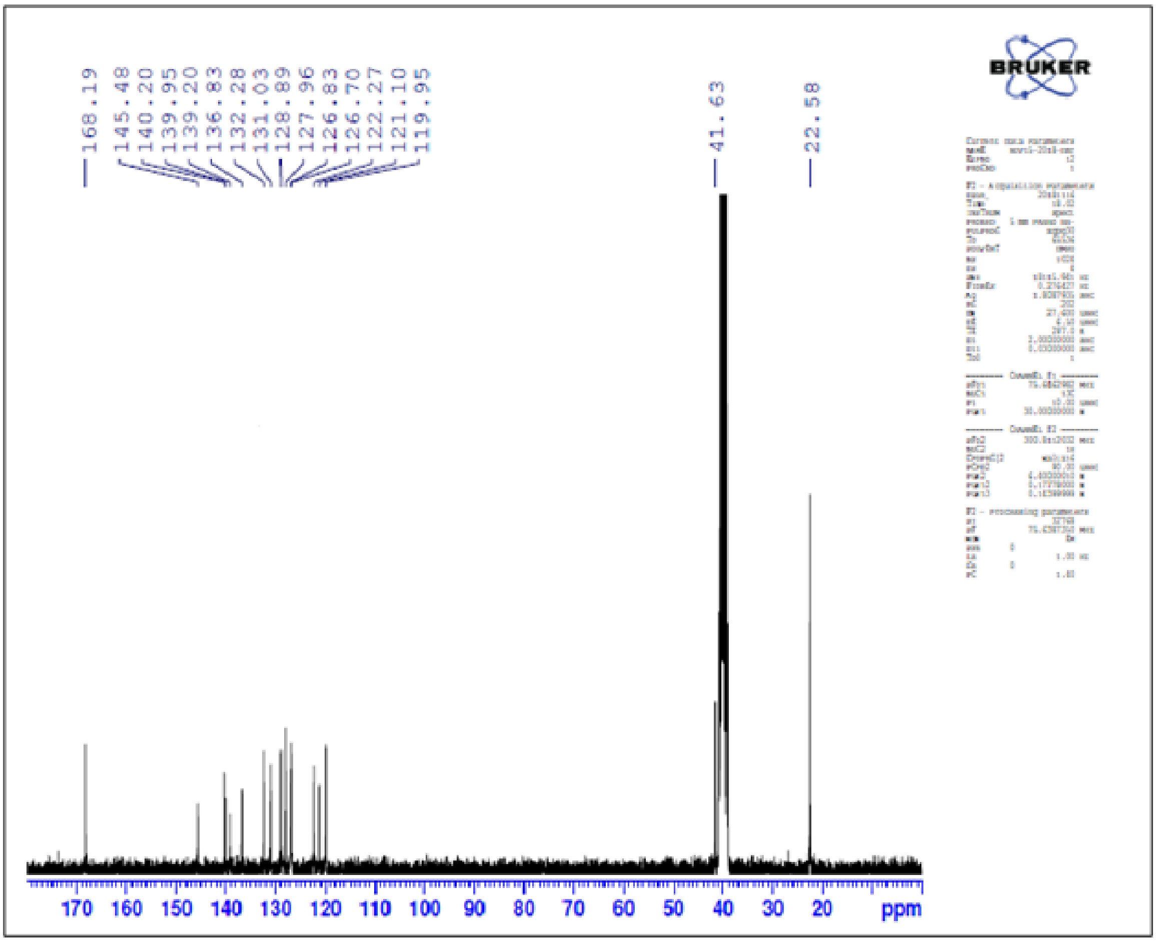

^{1}H NMR spectrum of compound **(3e)**

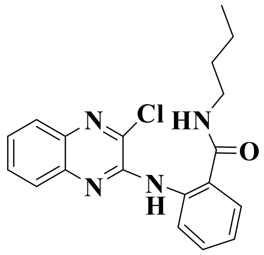

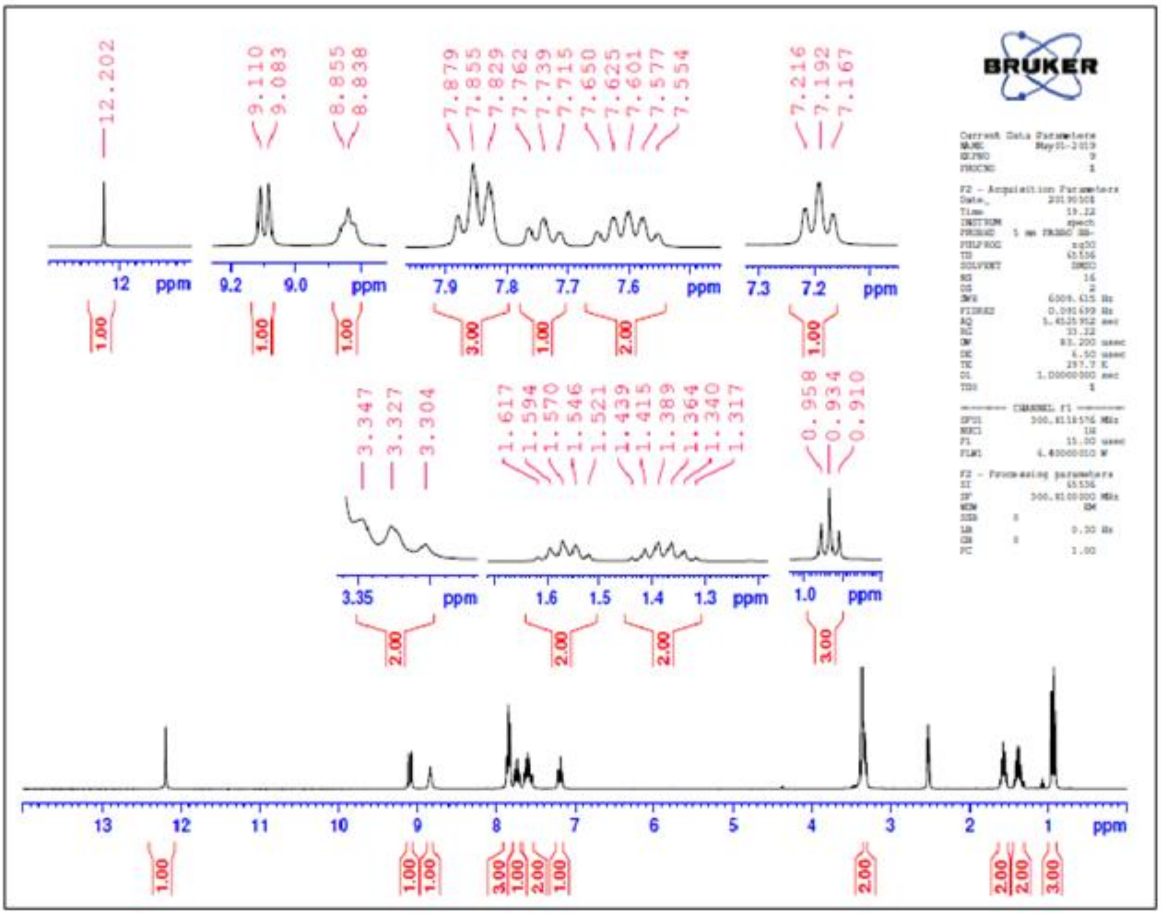

^{13}C NMR spectrum of compound **(3e)**

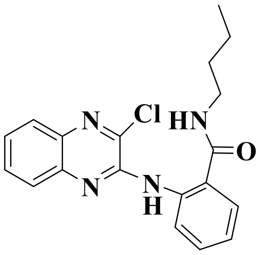

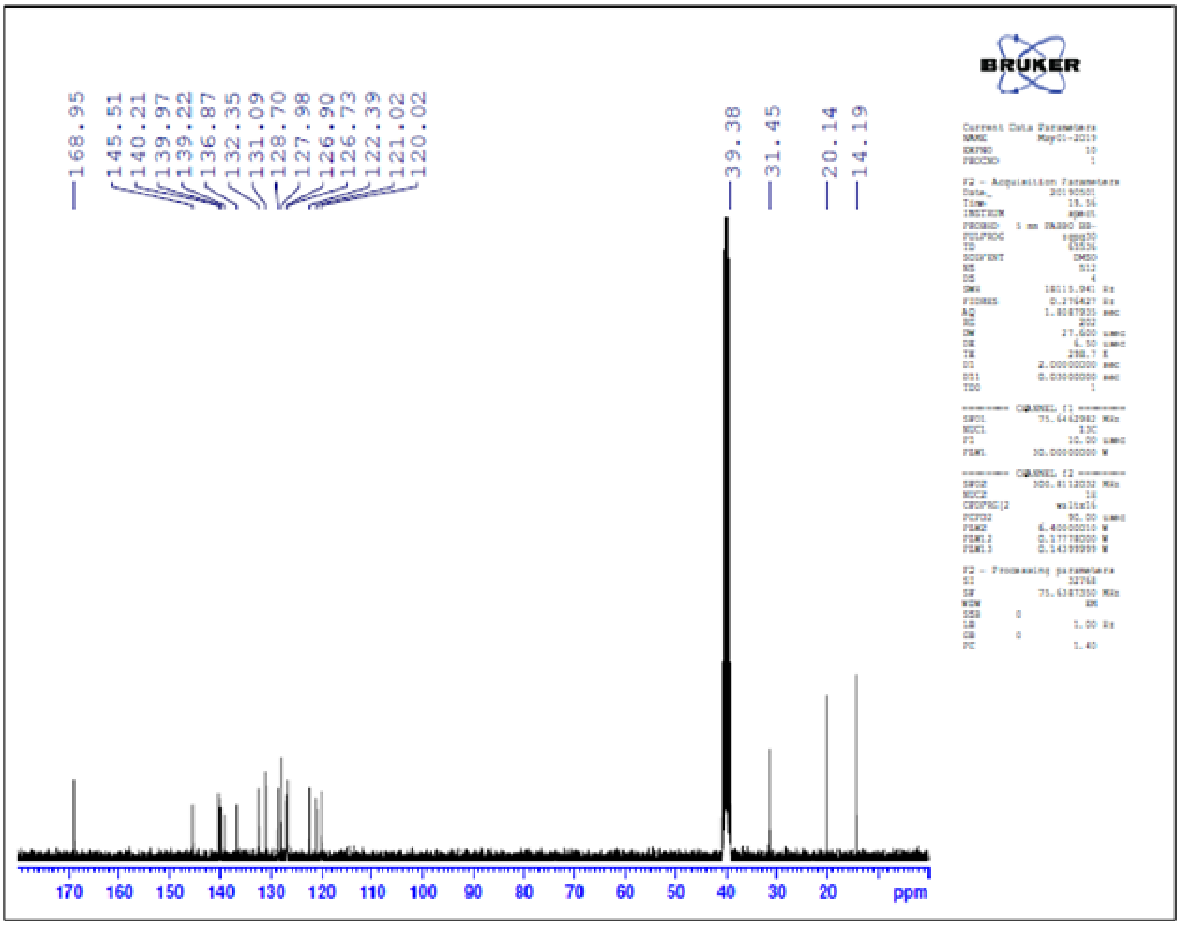

^{1}H NMR spectrum of compound **(3f)**

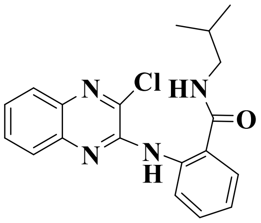

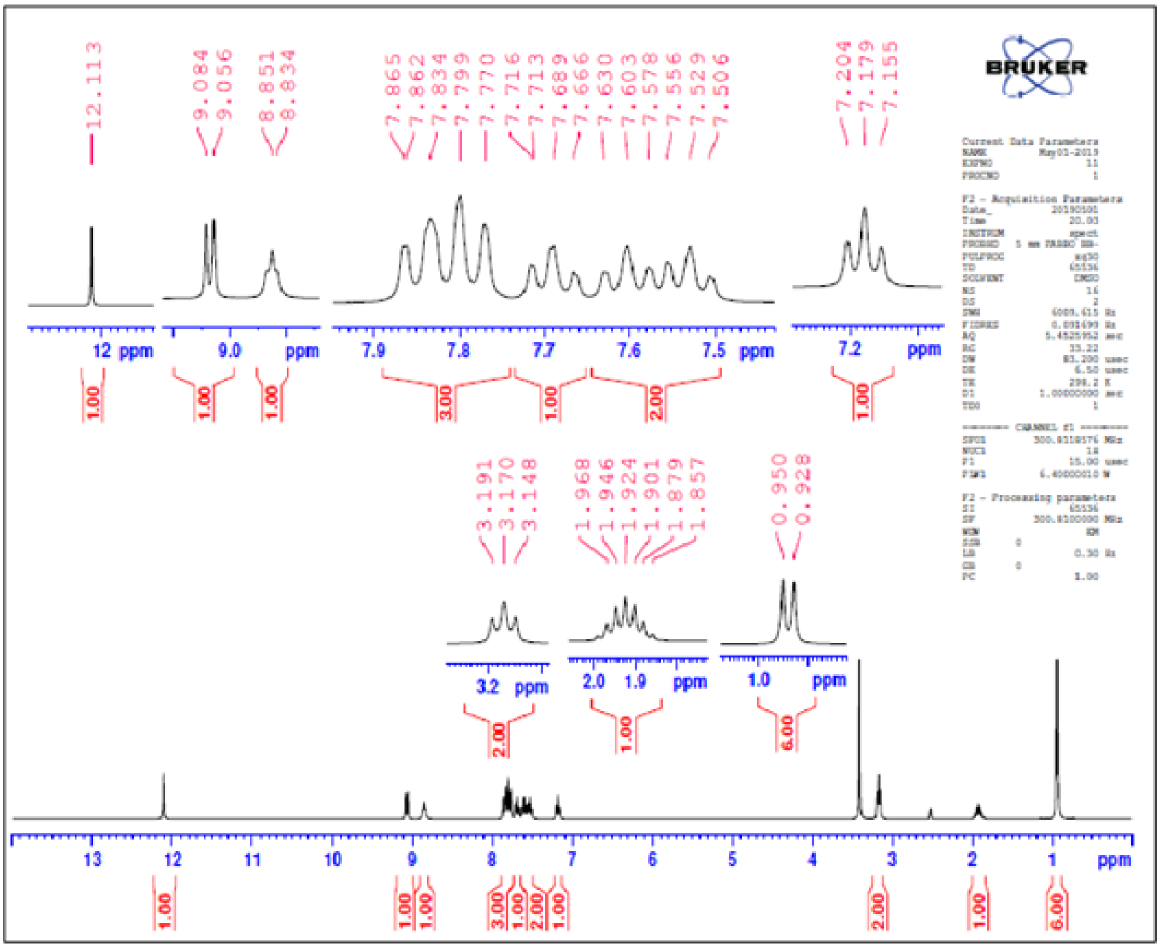

^{13}C NMR spectrum of compound **(3f)**

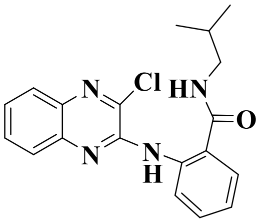

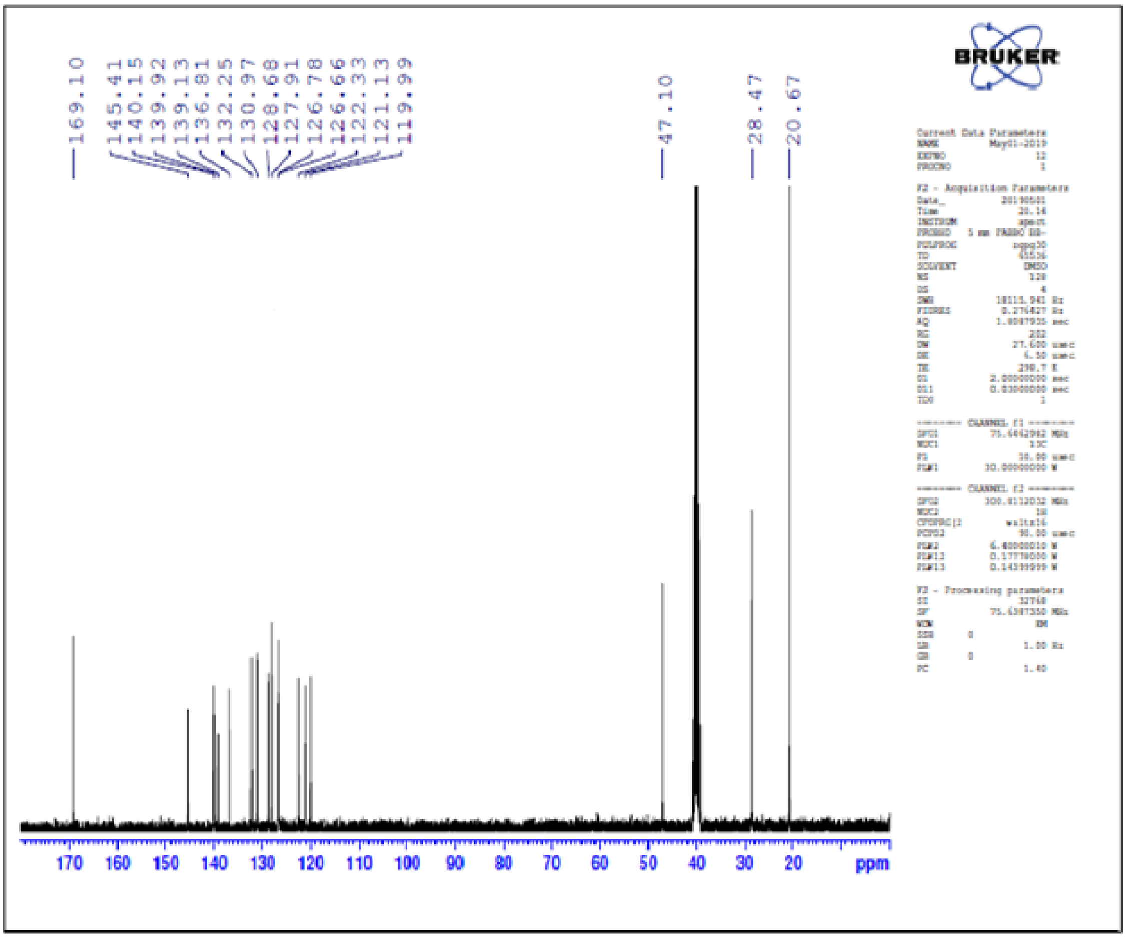

^{1}H NMR spectrum of compound **(3g)**

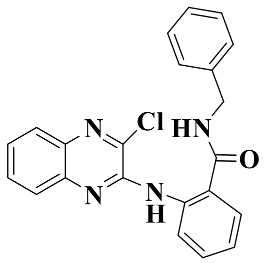

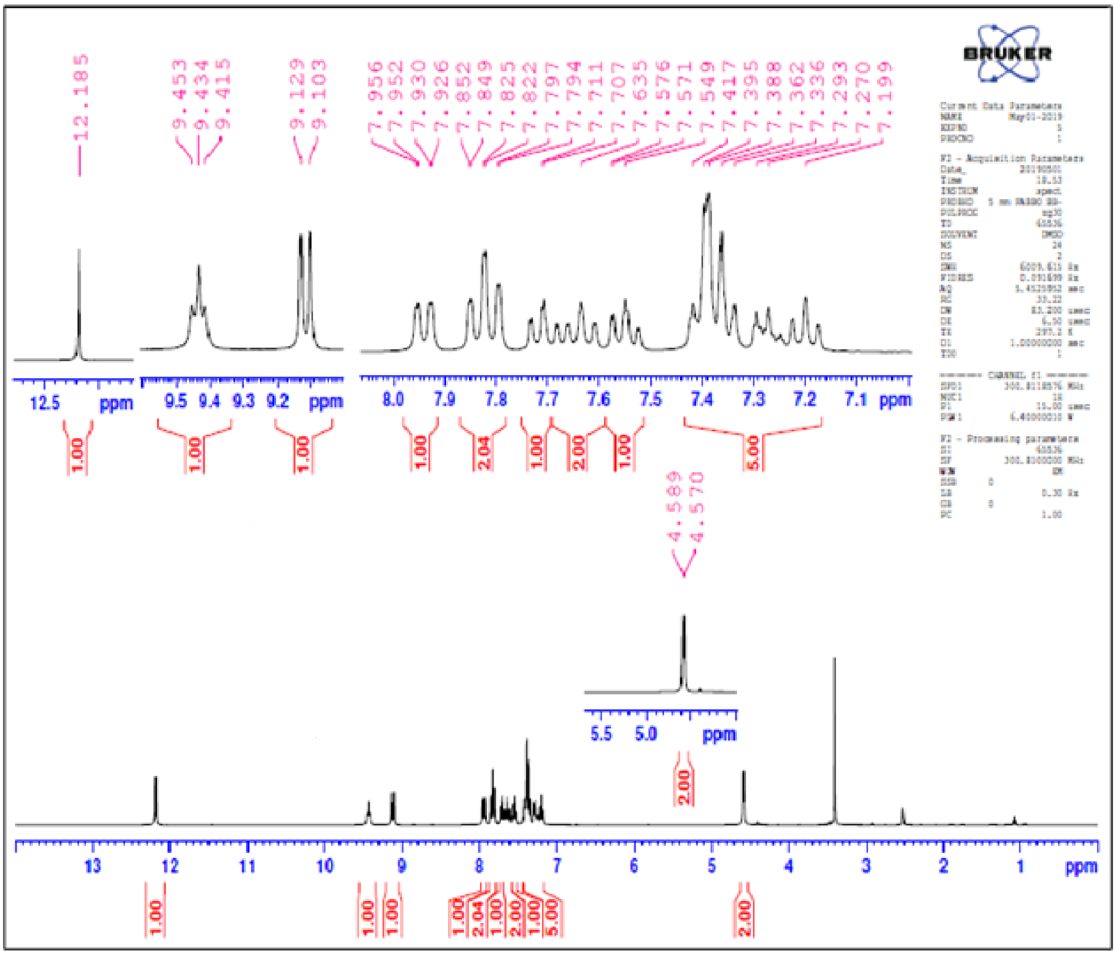

^{13}C NMR spectrum of compound **(3g)**

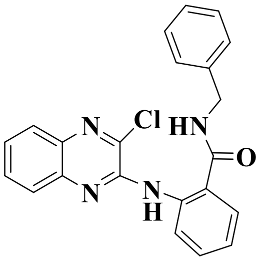

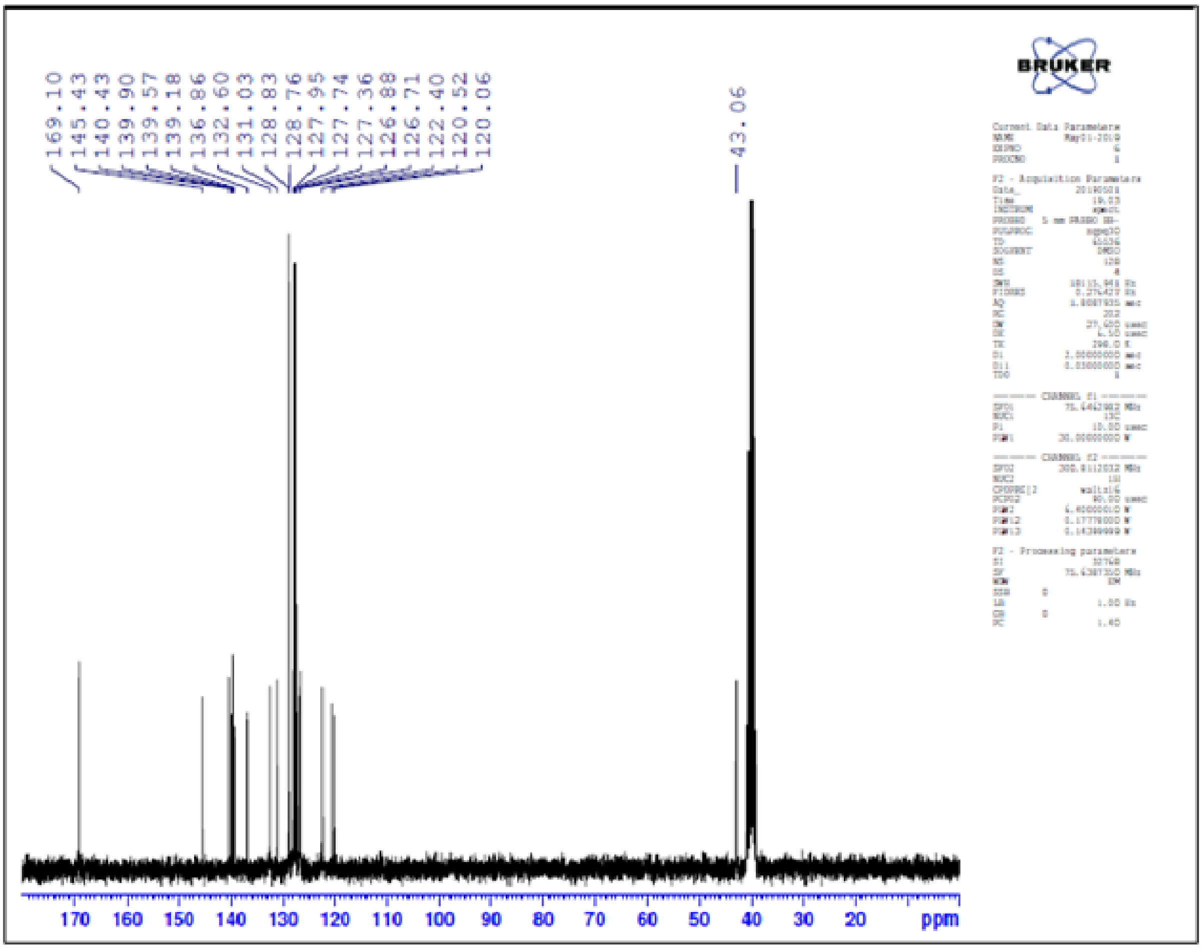

^{1}H NMR spectrum of compound **(4a)**

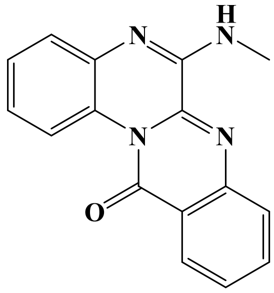

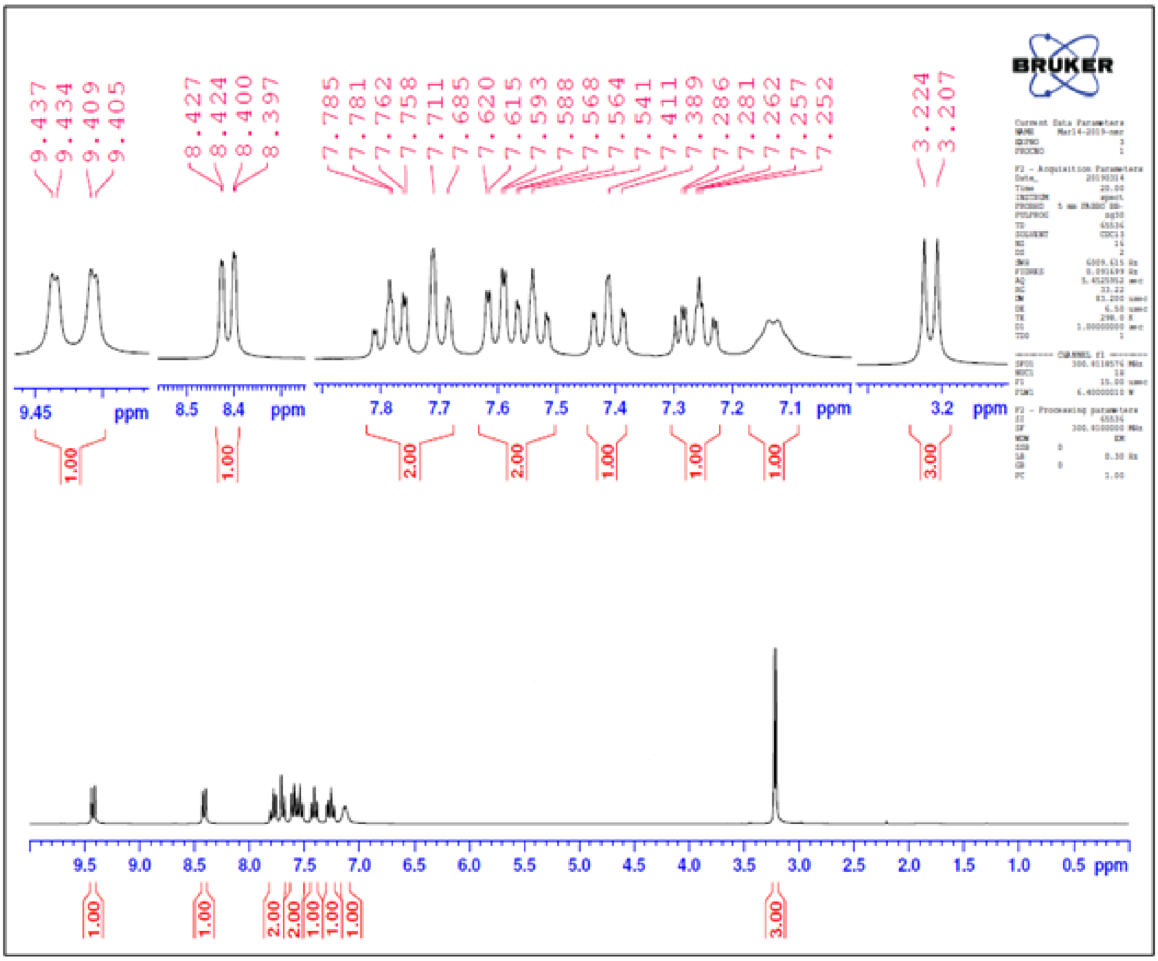

^{13}C NMR spectrum of compound **(4a)**

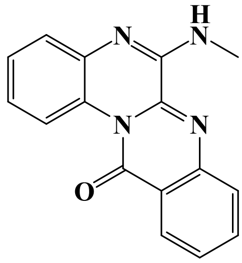

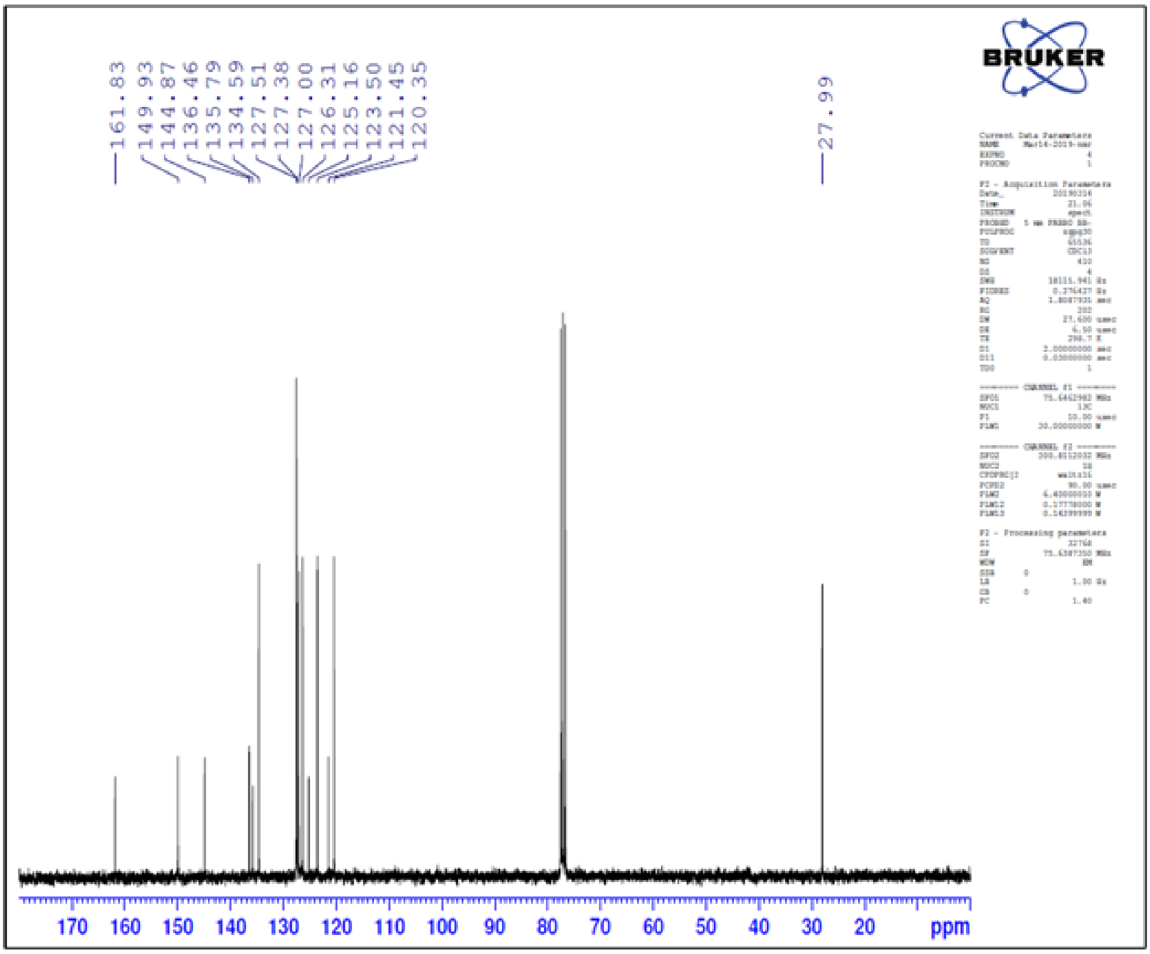

^{1}H NMR spectrum of compound **(4b)**

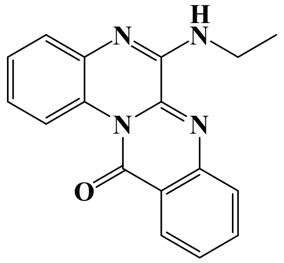

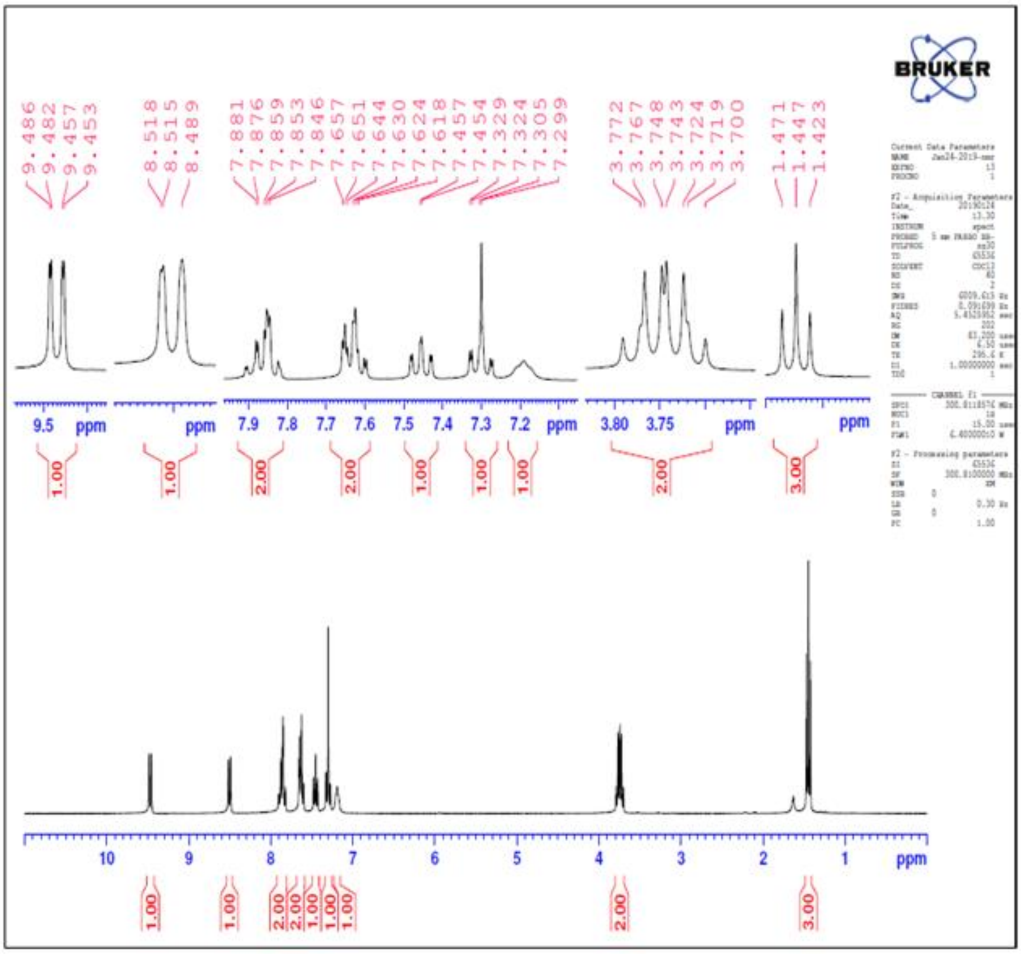

^{13}C NMR spectrum of compound **(4b)**

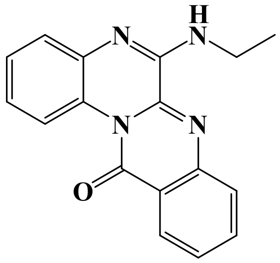

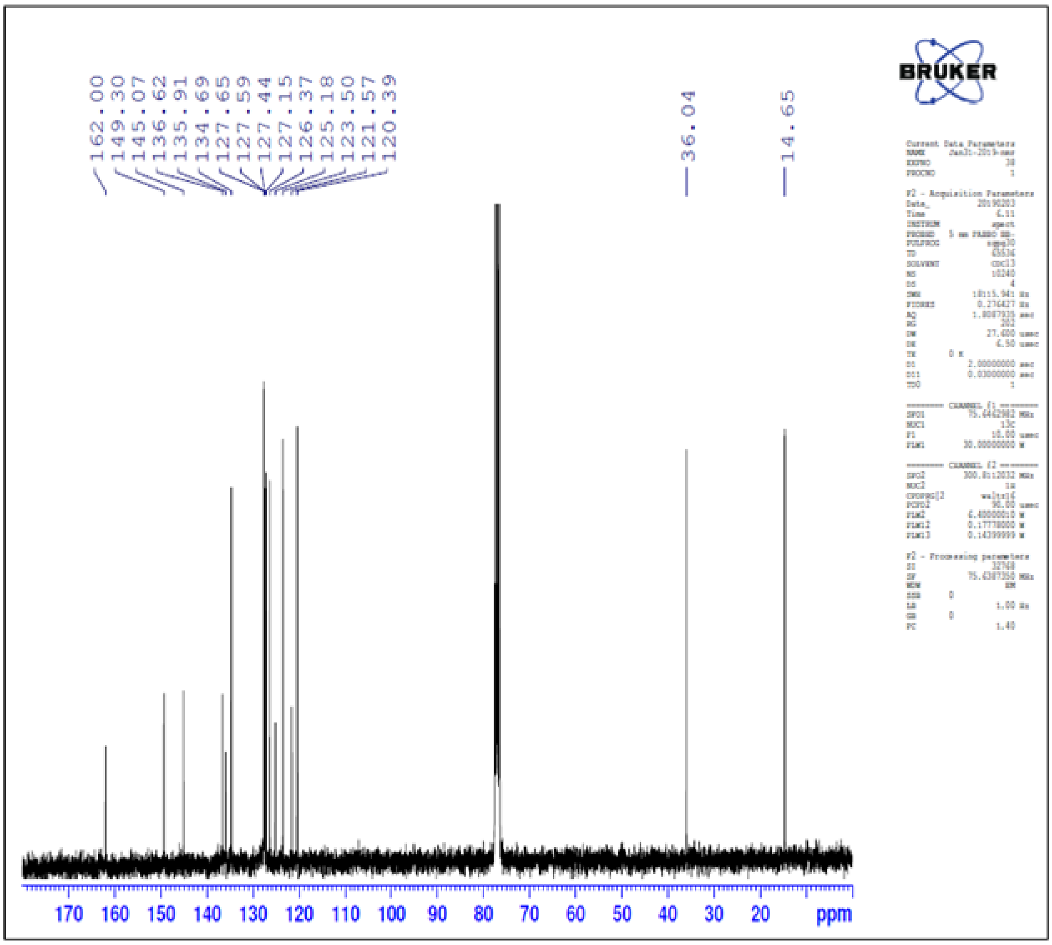

^{1}H NMR spectrum of compound **(4c)**

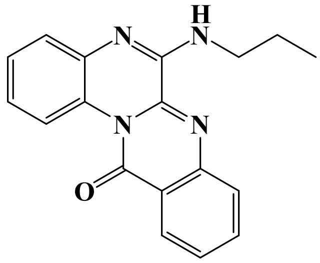

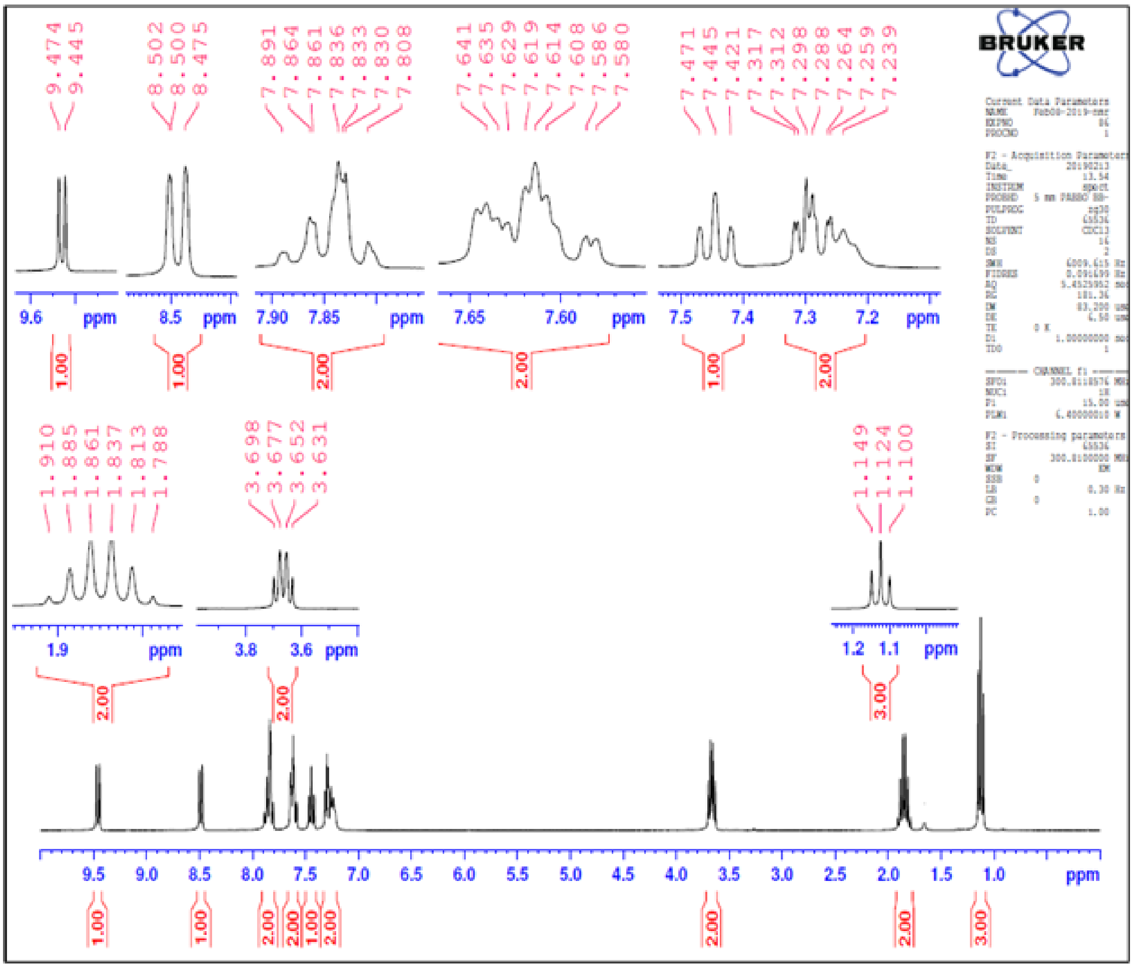

^{13}C NMR spectrum of compound **(4c)**

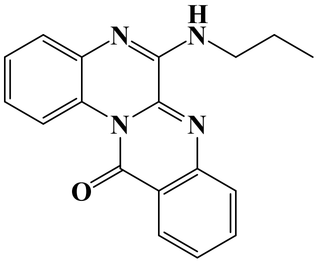

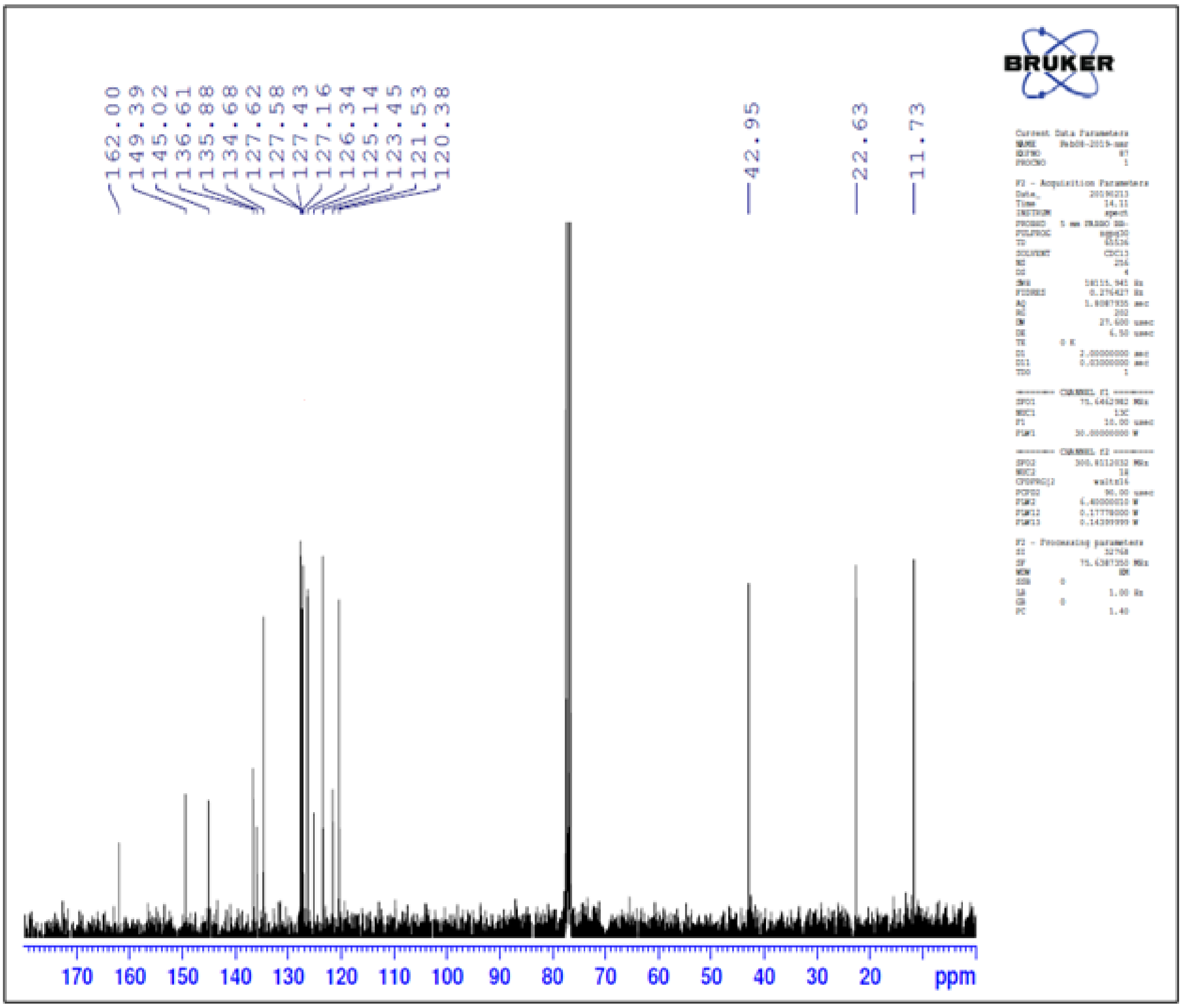

^{1}H NMR spectrum of compound **(4d)**

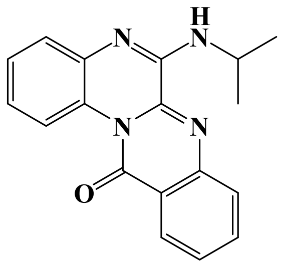

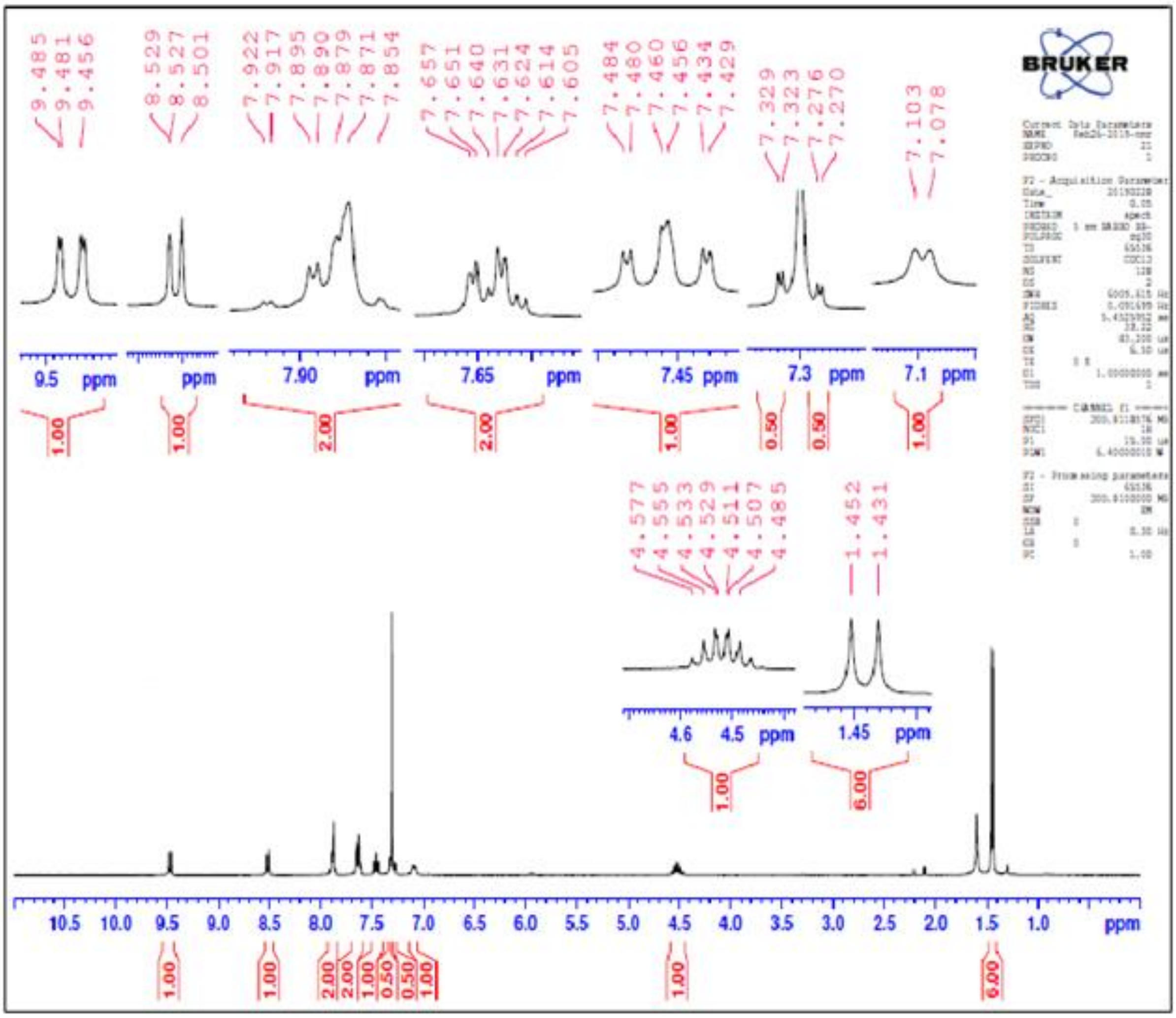

^{13}C NMR spectrum of compound **(4d)**

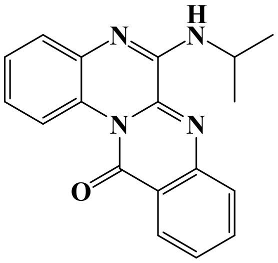

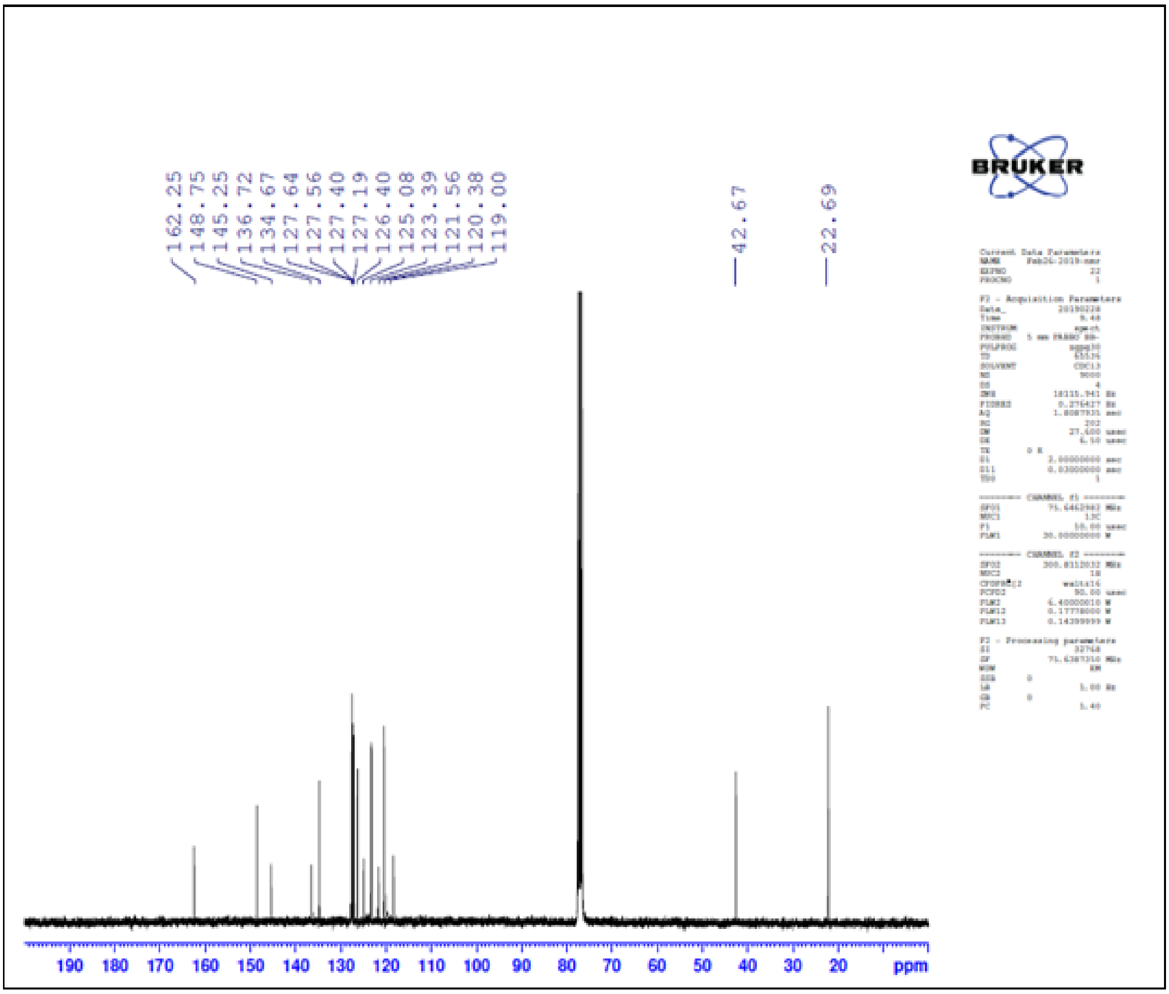

^{1}H NMR spectrum of compound **(4e)**

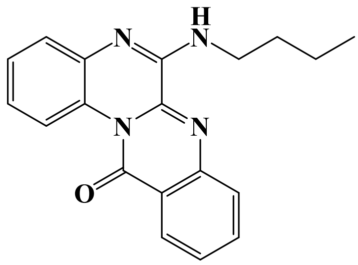

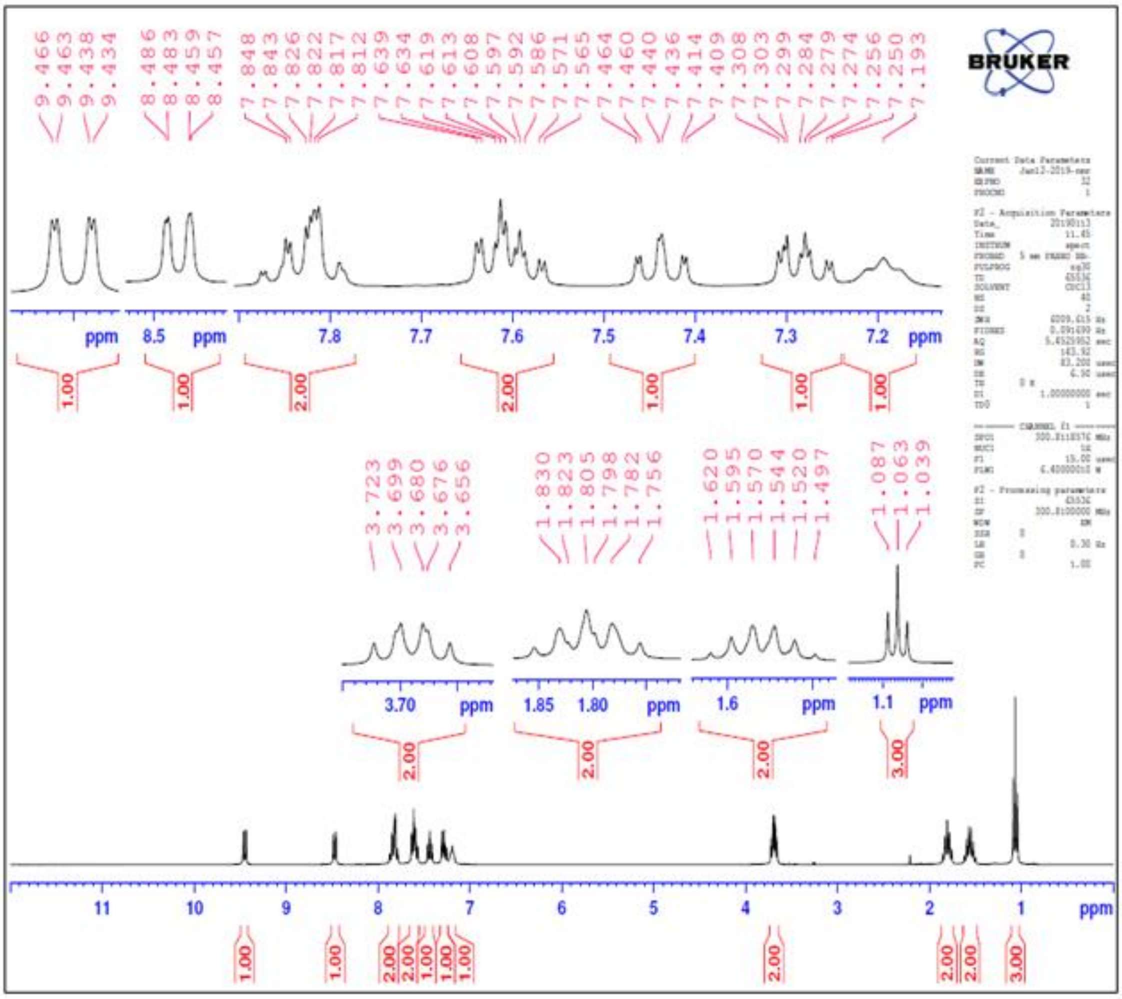

^{13}C NMR spectrum of compound **(4e)**

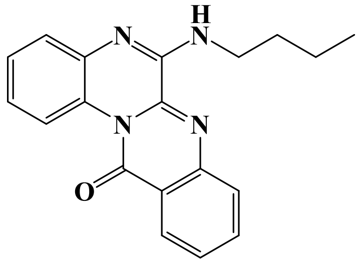

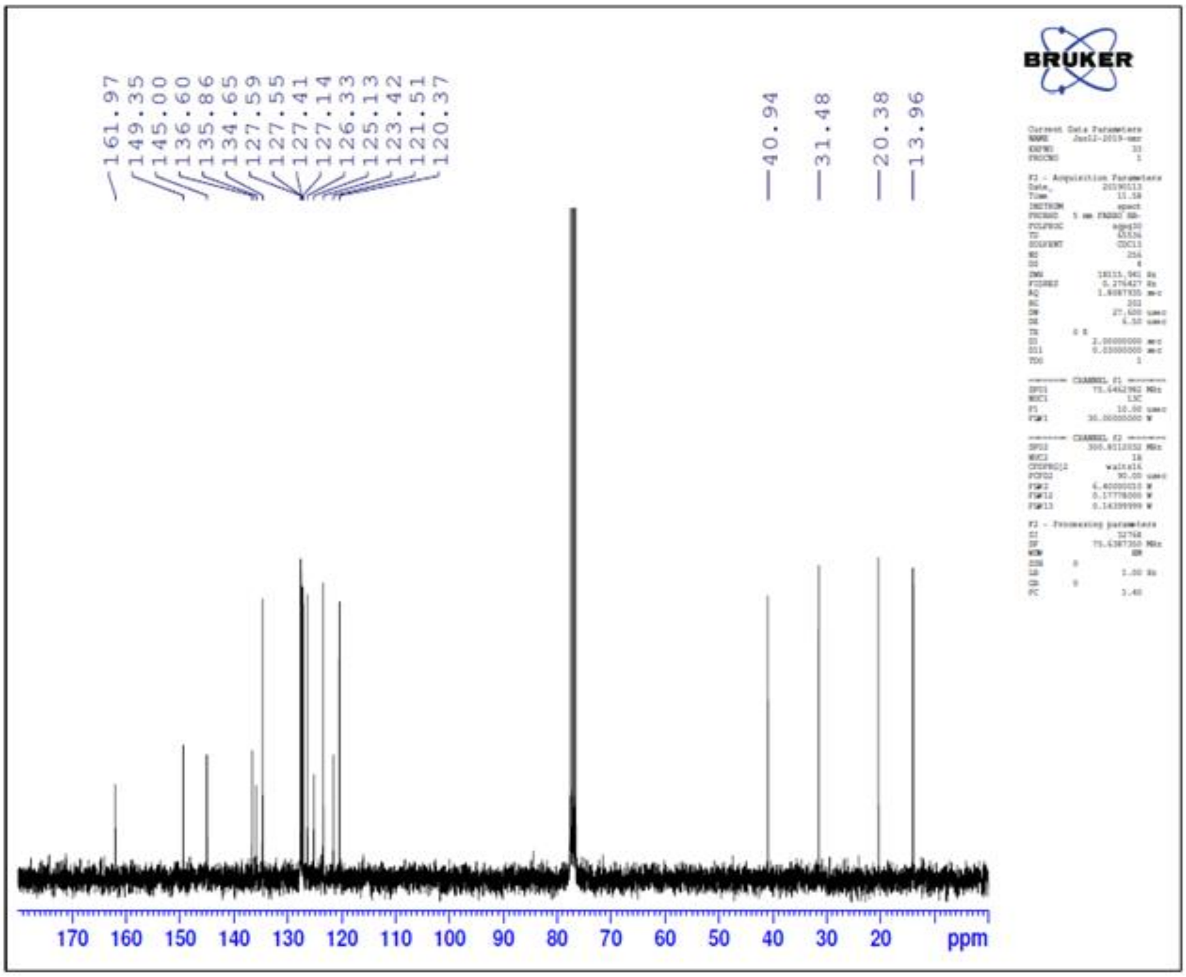

^{1}H NMR spectrum of compound **(4f)**

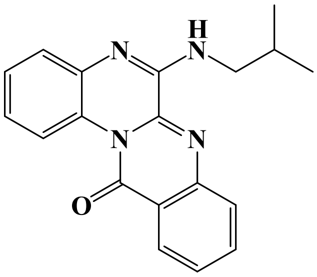

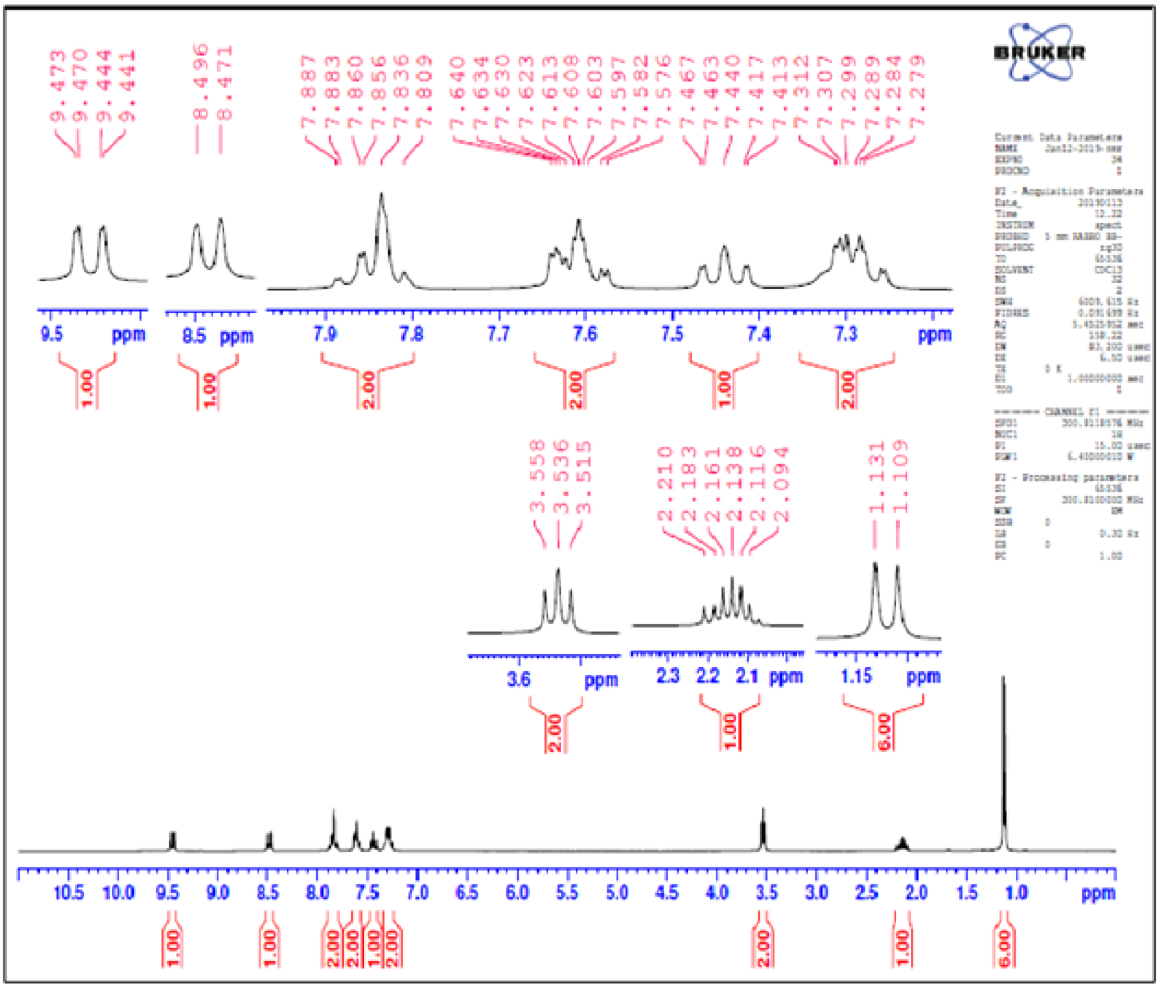

^{13}C NMR spectrum of compound **(4f)**

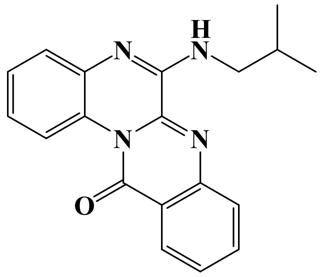

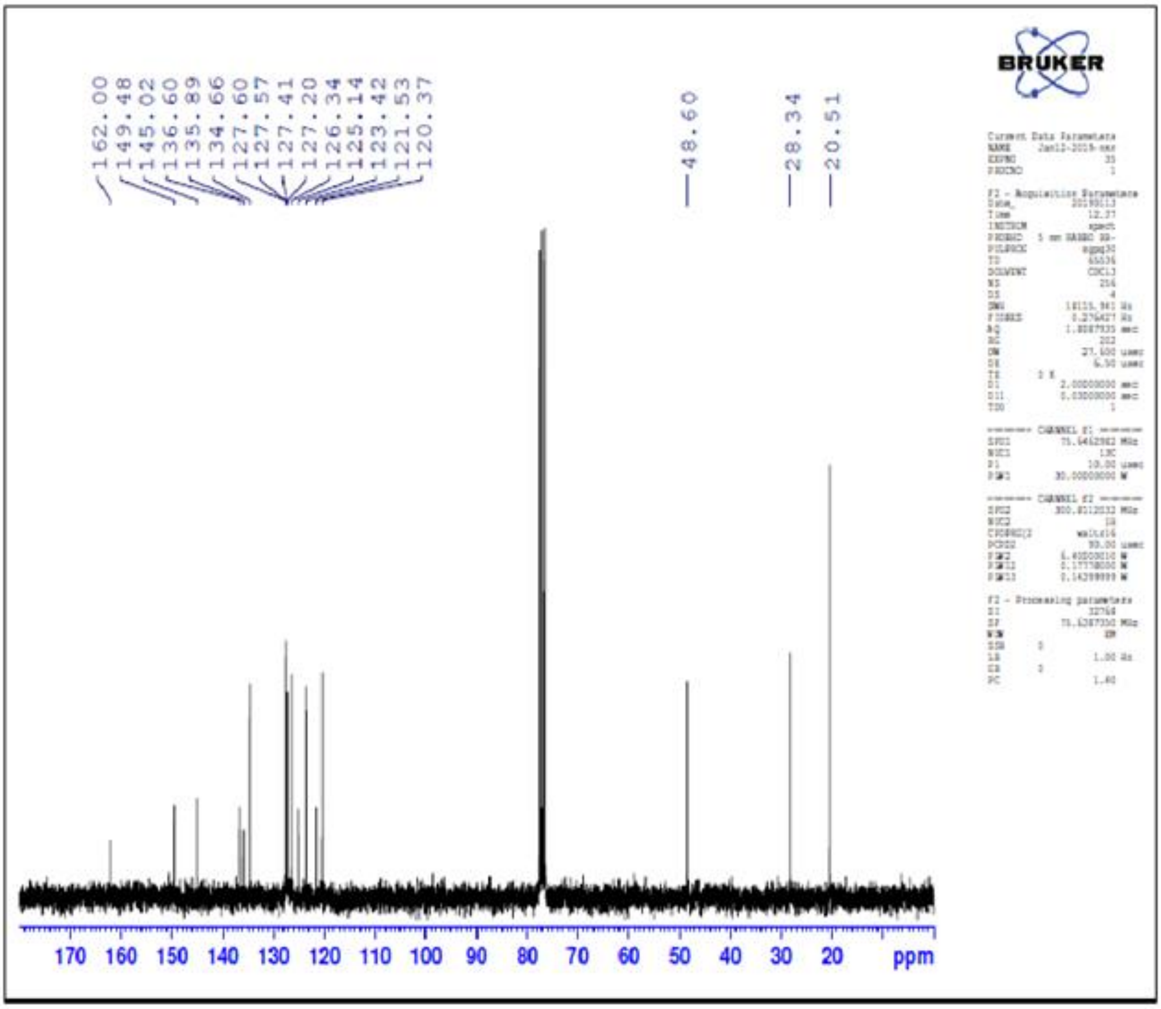

^{1}H NMR spectrum of compound **(4g)**

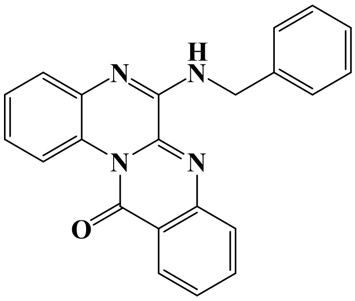

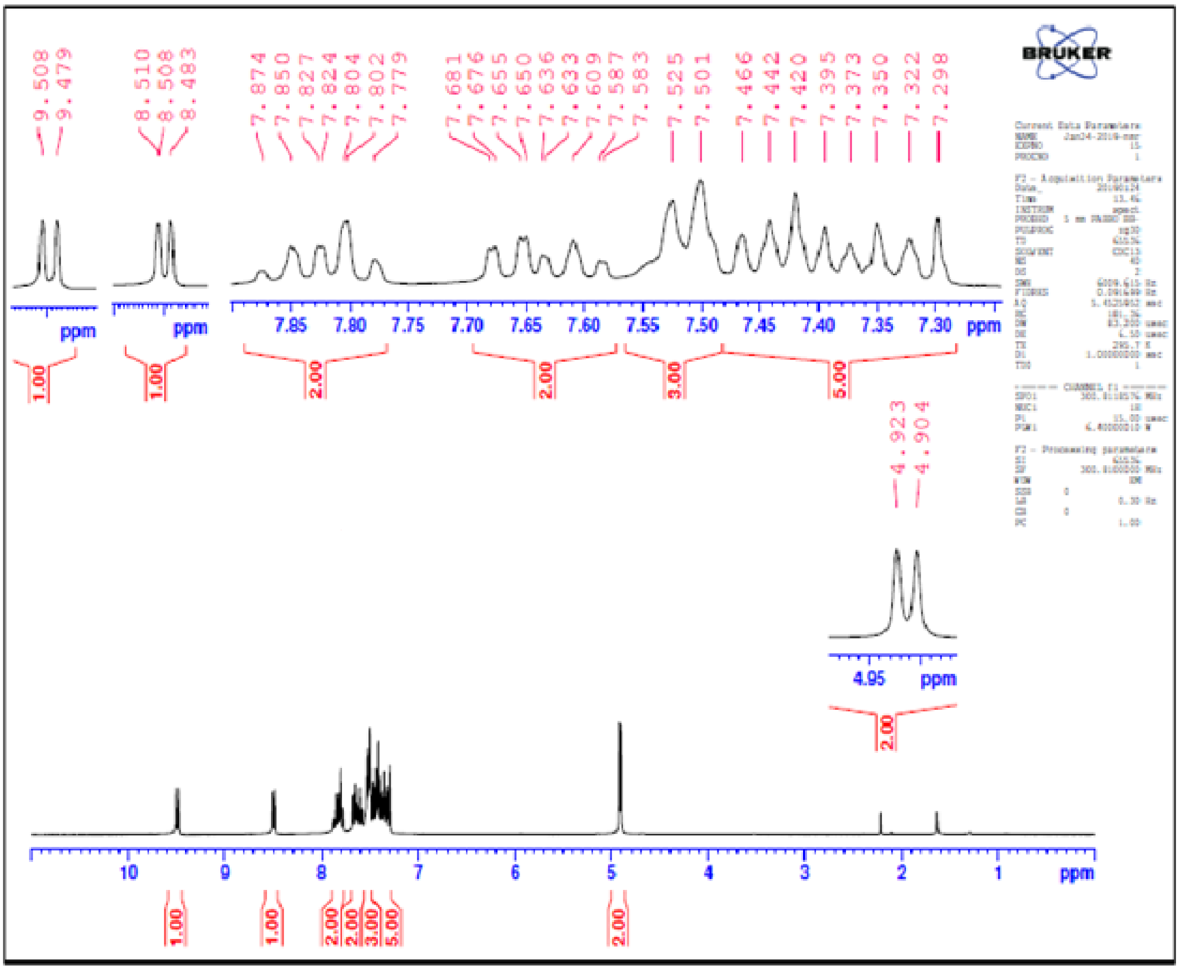

^{13}C NMR spectrum of compound **(4g)**

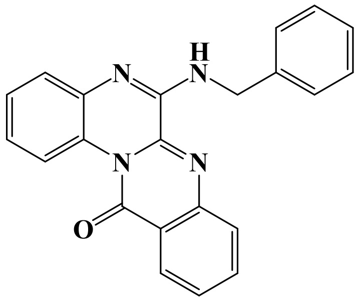

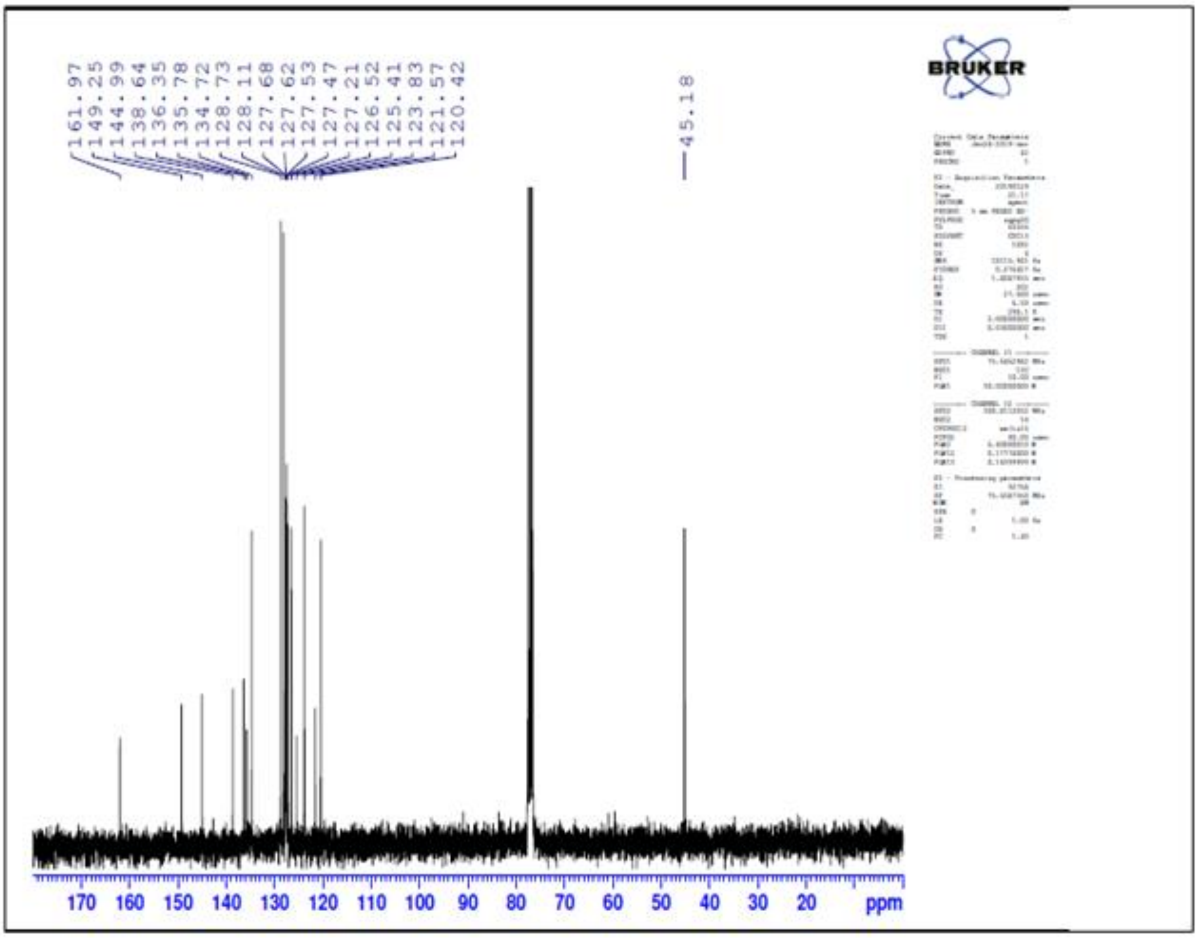

^{1}H NMR spectrum of compound **(4h)**

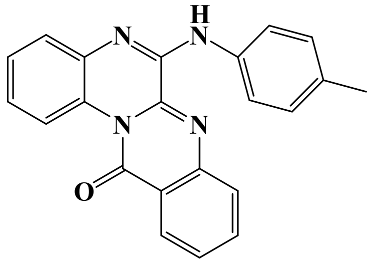

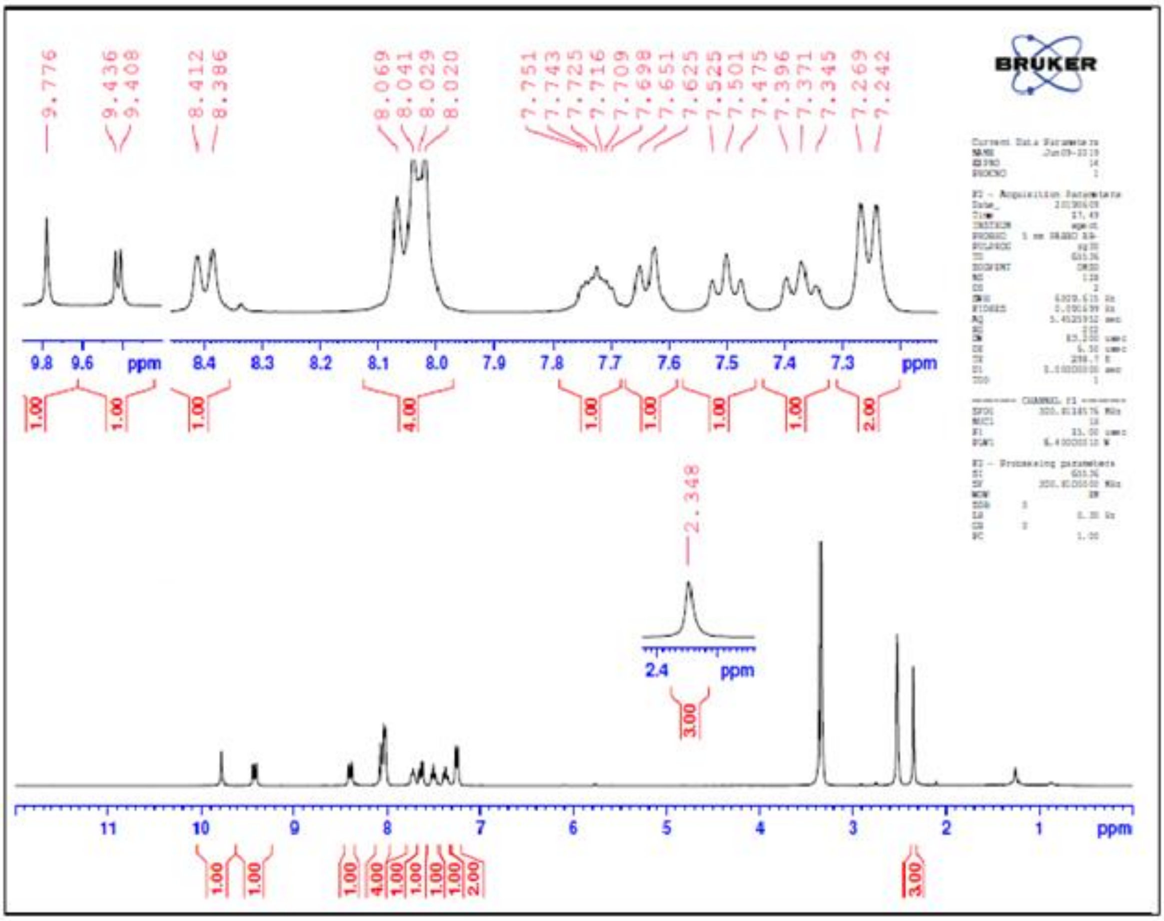

^{13}C NMR spectrum of compound **(4h)**

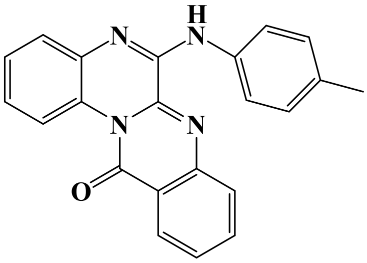

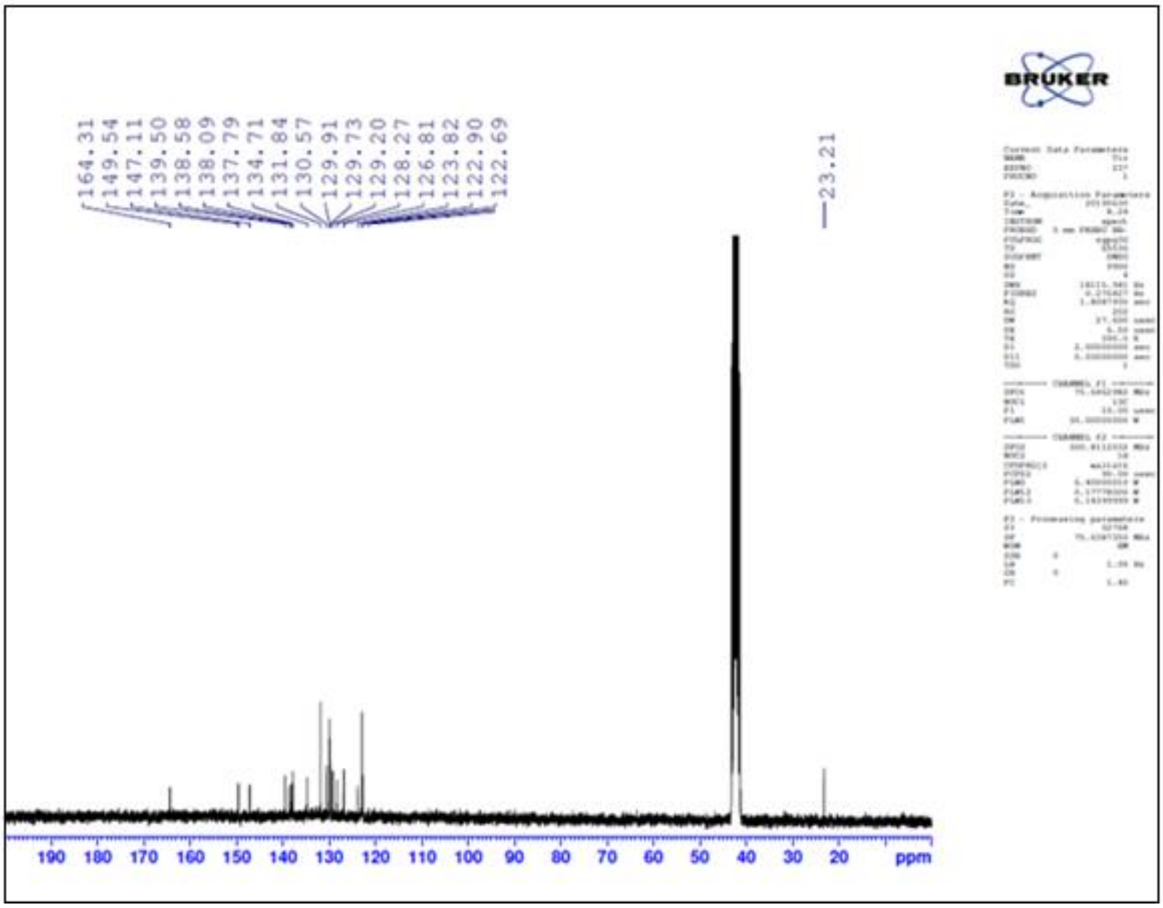

D_2O-exchangeable spectrum of compound **(4a)**

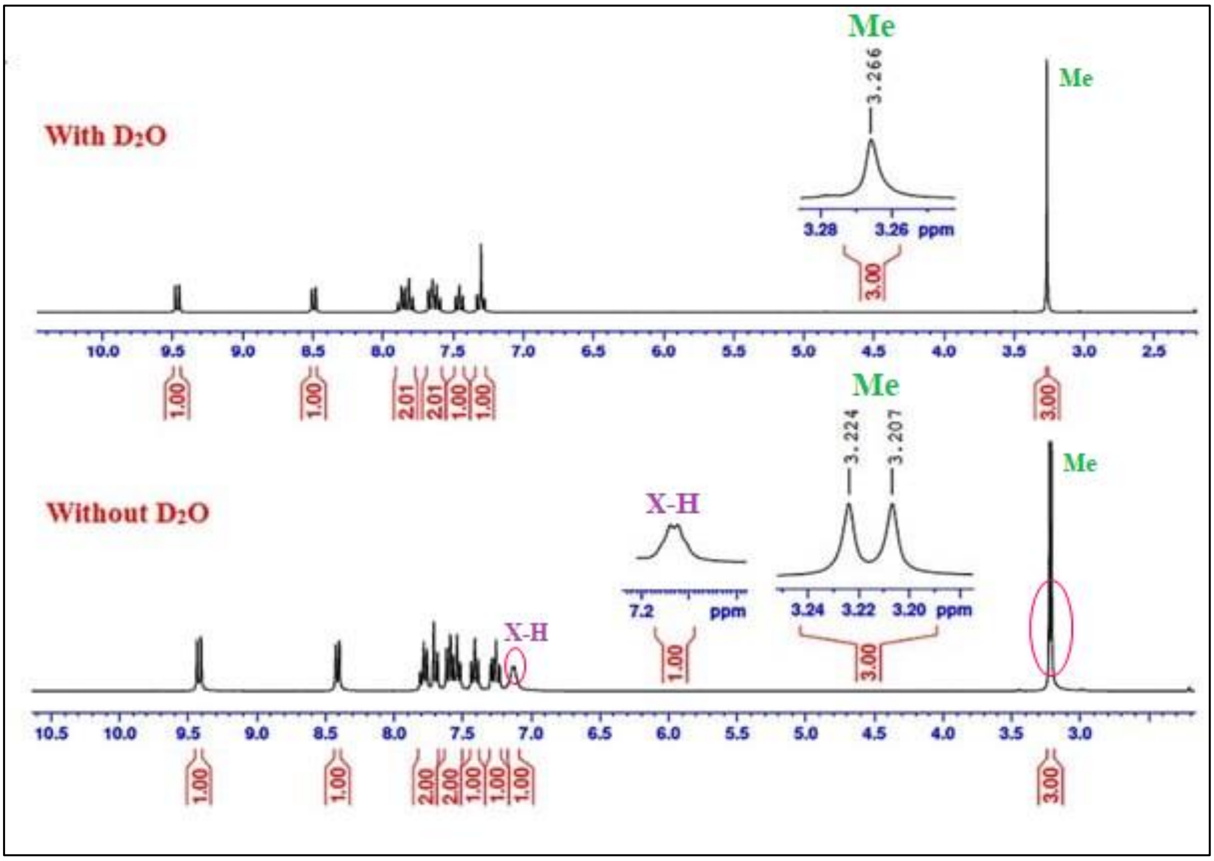

COSY spectrum of compound **(4a)**

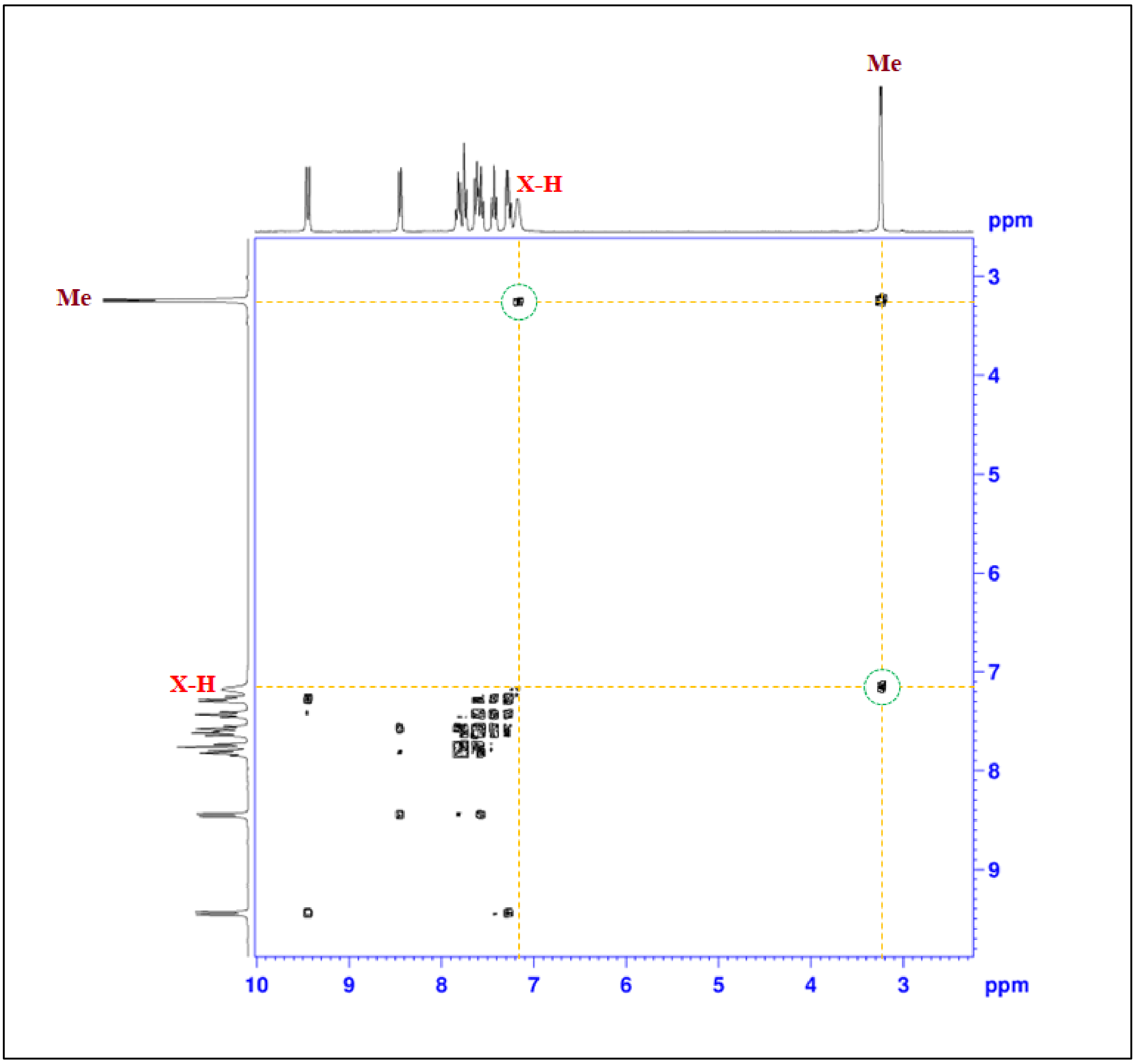

NOESY spectrum of compound **(4a)**

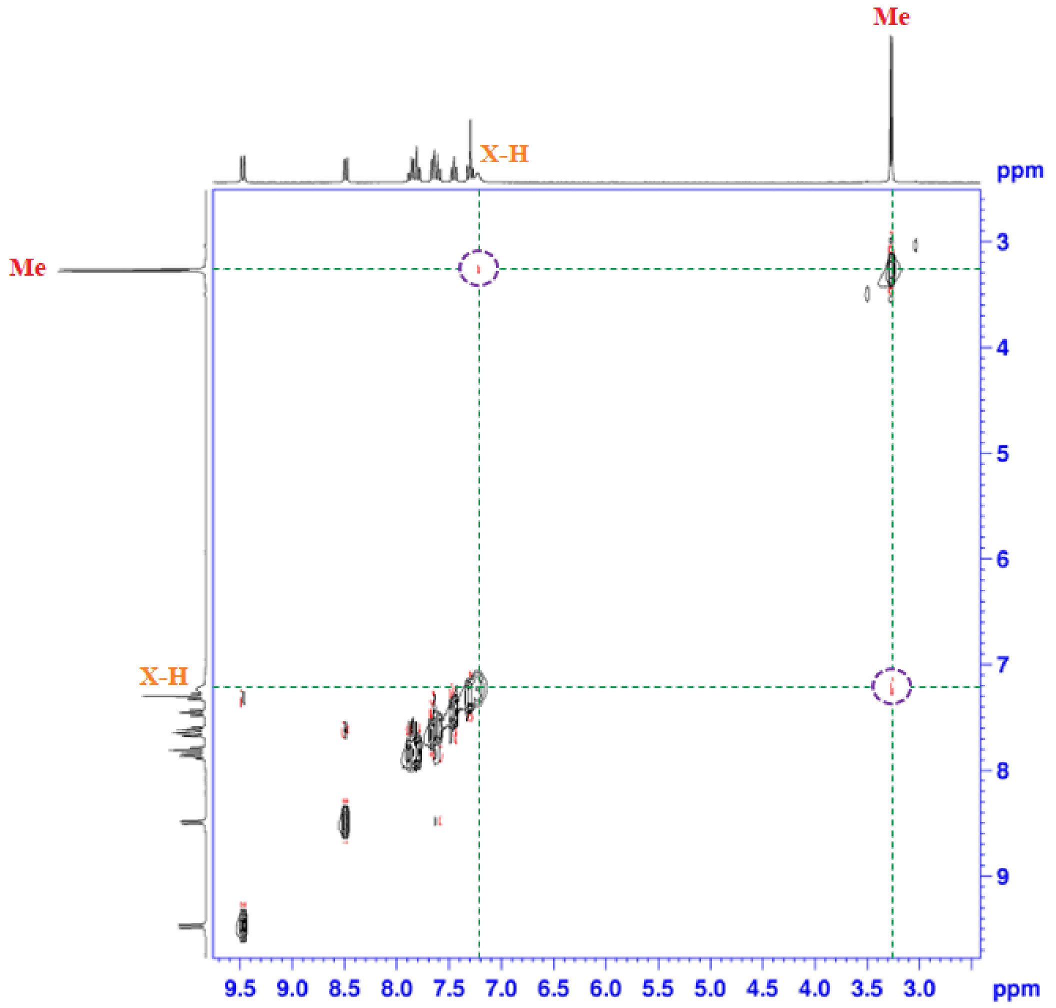

Crystallographic data

Single crystal X-ray data were collected on a Bruker KAPPA Apex II diffractometer equipped with an Apex II CCD detector, using Mo Kα X-ray radiation. The data were collected with APEX3[1] software; data reduction was performed using SAINT,[2] and corrected for absorption using SADABS[3] or TWINABS[4] with all programs implemented in the APEX3 suite.[1] The structures were solved by the intrinsic phasing method using SHELXT,[5] then refined with the assistance of SHELXL[5] employing ShelXle[6] as the graphic interface. All heavy atoms were refined anisotropically (hydrogen atoms were refined isotropically). Crystallographic report and CIF file for deposition in CCDC (deposition numbers 2357348)[7] were generated using FinalCIF software.[8] Ortep[9] style picture (Figures 6 in main document) was generated by CCDC Mercury[10] software used as a graphical front end to POV-Ray[11] rendering software. Default Mercury colors (light lilac for N, red for O, light green for Cl, grey for C, white for H) are used for atom color-coding. Ortep[9] plot (generated in PLATON)[12] of crystal structure is presented in Figures S1, crystal data and structure refinement is given in Table S1. Detailed crystallographic data and CIF file was deposited in The Cambridge Crystallographic Data Centre with number CCDC 2357348; these data can be obtained free of charge via www.ccdc.cam.ac.uk/data_request/cif.

Table S1. Crystallographic data.

Compound	**4e perchlorate**
CCDC number	2357348
Chemical Formula	$C_{19}H_{19}ClN_4O_5$
Formula Weight	418.83
Temperature/ K	100(2)
Crystal system	monoclinic
Space group	$P2_1/n$ (14)
a/ Å	8.416(3)
b/ Å	14.747(5)
c/ Å	15.257(6)
α/ °	90
β/ °	103.503(8)
γ/ °	90

V/ Å^3	1841.3(12)
Z	4
ρ_{calc}/g cm^{-3}	1.511
μ(Mo-Kα)/ mm^{-1}	0.250
F(000)	872
Crystal size/ mm	0.174×0.151×0.139
Theta range for data collection/ °	3.89 to 56.60 (0.75 Å
Index range	−11 ≤ h ≤ 11<br>−19 ≤ k ≤ 19<br>−20 ≤ l ≤ 20
Reflections collected	25247
Independent reflections	4580
Data / restraints / parameters	4580/0/271
R_{int}	0.0831
Final R indices [I>2sigma(I)]	R_1 = 0.0453<br>wR_2 = 0.0888
R indices (all data)	R_1 = 0.0918<br>wR_2 = 0.1053
GooF on F^2	1.005

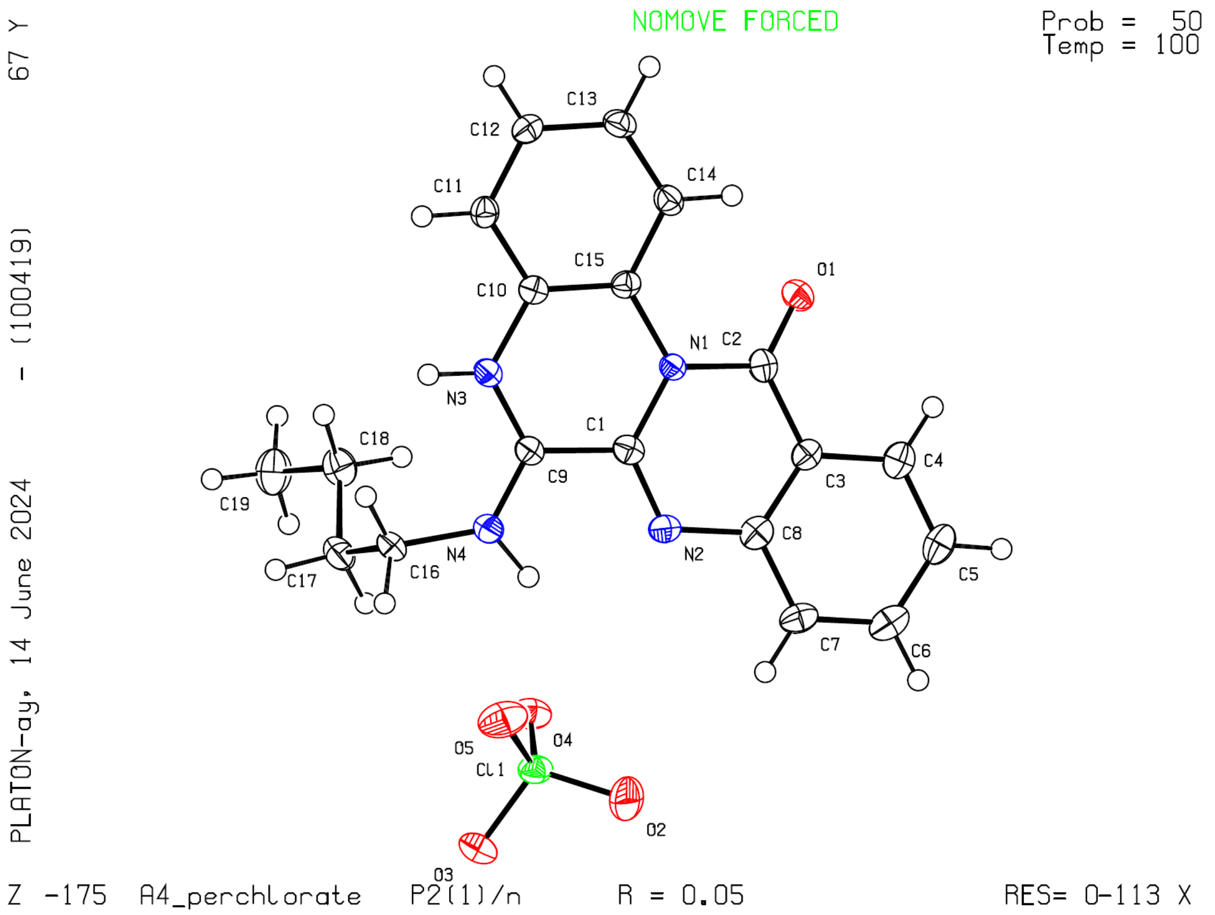

Figure S1. Molecular structure of 4e perchlorate salt; thermal ellipsoids are shown for 50% probability.

(1) APEX3, Bruker AXS Inc., Madison, WI, 2016.

(2) SAINTPlus, Bruker AXS Inc., Madison, WI, 2012.

(3) SADABS, Bruker AXS Inc., Madison, WI, 2012.

(4) TWINABS, Bruker AXS Inc., Madison, WI, 2001, 2012.

(5) Sheldrick, G. SHELXT - Integrated space-group and crystal-structure determination. *Acta Crystallographica Section A* **2015**, *71*, 3. doi:10.1107/S2053273314026370

(6) Hubschle, C. B.; Sheldrick, G. M.; Dittrich, B. ShelXle: a Qt graphical user interface for SHELXL. *J. Appl. Crystallogr.* **2011**, *44*, 1281. doi:10.1107/S0021889811043202

(7) Groom, C. R.; Bruno, I. J.; Lightfoot, M. P.; Ward, S. C. The Cambridge Structural Database. *Acta Crystallographica Section B* **2016**, *72*, 171. doi:10.1107/S2052520616003954

(8) Kratzert, D., FinalCIF, 2022.

(9) Farrugia, L. WinGX and ORTEP for Windows: an update. *J. Appl. Crystallogr.* **2012**, *45*, 849. doi:10.1107/S0021889812029111

(10) Macrae, C. F.; Sovago, I.; Cottrell, S. J.; Galek, P. T. A.; McCabe, P.; Pidcock, E.; Platings, M.; Shields, G. P.; Stevens, J. S.; Towler, M.; Wood, P. A. Mercury 4.0: from visualization to analysis, design and prediction. *J. Appl. Crystallogr.* **2020**, *53*, 226. doi:10.1107/S1600576719014092

(11) Persistence of Vision Pty. Ltd. (2004), Persistence of Vision Raytracer (Version 3.7.0) [Computer software]. Retrieved from http://www.povray.org/download/, 2013.

(12) Spek, A. Structure validation in chemical crystallography. *Acta Crystallographica Section D* **2009**, *65*, 148. doi:10.1107/S090744490804362X
